# Degradation-constrained multi-agent reinforcement learning with centralized training and decentralized execution for vehicle-to-grid optimization in renewable-dominated distribution networks

**DOI:** 10.1038/s41598-026-55471-3

**Published:** 2026-06-03

**Authors:** Yeshitela Shiferaw, Mehari Kiros, Kumlachew Yeneneh, Gadisa Sufe

**Affiliations:** 1 Department of Electrical Engineering, College of Engineering, Ethiopian Defense University, P.O. Box 1041, Bishoftu, Ethiopia; 2 Department of Computer Science and Engineering, College of Engineering, Ethiopian Defence University, P.O. Box 1041, Bishoftu, Ethiopia; 3 Department of Motor Vehicle Engineering, College of Engineering, Ethiopian Defence University, P.O. Box 1041, Bishoftu, Ethiopia; 4 Faculty of Mechanical Engineering, Wrocław University of Science and Technology, Wrocław, 50-370 Poland

**Keywords:** Vehicle to grid optimization, Multi agent reinforcement learning, Battery degradation modeling, Renewable energy integration, Distribution network stability, Energy science and technology, Engineering, Mathematics and computing

## Abstract

High renewable penetration and large-scale electric vehicle integration impose voltage instability, frequency deviation, and network congestion challenges in distribution systems while accelerating battery degradation. This study proposes a degradation constrained multi agent reinforcement learning framework based on centralized training with decentralized execution for coordinated vehicle to grid optimization. The method integrates DC power flow constraints, stochastic renewable uncertainty, and electrochemical battery aging dynamics within a unified control architecture to ensure network aware and lifecycle aware scheduling. The framework is evaluated on the IEEE 33 bus distribution network with electric vehicle penetration up to 50% and compared against two baselines: a rule based conventional V2G scheduler and a single agent deep reinforcement learning controller without coordinated network constraints or degradation penalization. Grid stability is quantified using a normalized composite index derived from frequency and voltage magnitude deviations. Across repeated simulation trials, the proposed approach improves the mean stability index by approximately 10% relative to the rule-based method and 7% relative to the single agent baseline. Renewable utilization increases by about 18% age points, peak load reduction reaches 40% under high penetration scenarios, and cumulative battery aging decreases by nearly 13% over a 24-hour horizon, demonstrating enhanced coordinated control performance within standardized simulation environments.

## Introduction

The accelerating global transition toward low-carbon energy systems has led to a rapid increase in the integration of renewable energy sources (RES), particularly solar and wind, into modern power grids^1,2^. Although higher renewable penetration improves sustainability and reduces emissions, the inherent intermittency and stochastic nature of these sources introduce significant operational challenges, including frequency instability, voltage fluctuations, and increased balancing requirements^3^. As a result, flexible distributed energy resources have become essential for maintaining reliable grid operation under high renewable penetration scenarios^4^.

In this evolving energy landscape, electric vehicles (EVs), supported by advances in battery technology and decarbonization policies, are increasingly viewed not only as transportation assets but also as mobile energy storage units^5^. Vehicle-to-grid (V2G) technology enables bidirectional power exchange between EV batteries and grid infrastructure, allowing aggregated EV fleets to operate as distributed storage systems capable of supporting peak shaving, frequency regulation, demand response, and renewable energy buffering. Recent studies indicate that, when effectively coordinated within distribution network environments, V2G systems can substantially enhance grid flexibility and resilience. However, the growing complexity associated with large-scale EV fleets, renewable generation variability, and real-time grid constraints necessitates advanced optimization frameworks capable of managing multidimensional and highly dynamic operational conditions^6,7^.

Extensive research in power systems engineering has explored V2G integration frameworks focusing on coordinated charging strategies, distributed energy resource aggregation, and grid stability enhancement^8^. Conventional optimization techniques, including stochastic programming, model predictive control, and heuristic scheduling algorithms, have demonstrated effectiveness in load balancing and participation in energy markets^9^. Nevertheless, these approaches often rely on simplified assumptions regarding demand profiles or user behavior, limiting scalability and adaptability in highly dynamic environments^10^. Recent reviews emphasize that future V2G deployment requires adaptive control mechanisms capable of handling uncertainty, real-time data streams, and large-scale distributed coordination^11^. In addition to technical challenges, integration efforts must also address cybersecurity risks, regulatory constraints, and interoperability across heterogeneous grid infrastructures^12,13^.

Artificial intelligence (AI) and machine learning (ML) have emerged as promising tools for addressing nonlinear system dynamics and uncertainty in distribution network environments^14^. Recent studies demonstrate the application of deep learning, reinforcement learning, and hybrid AI frameworks for energy forecasting, adaptive scheduling, and decentralized control of distributed energy assets^15^. For example, reinforcement learning-based V2G coordination strategies have shown the potential to reduce peak loads, optimize energy flows, and improve operational efficiency under uncertain renewable conditions^16^. Similarly, AI-driven V2G management frameworks that incorporate semantic awareness, edge computing, and intelligent prioritization mechanisms have demonstrated measurable improvements in response time and energy distribution efficiency compared with traditional rule-based systems^10,17^. Parallel developments in AIoT architectures further enable real-time monitoring and predictive control by combining IoT sensing with data-driven optimization algorithms, thereby enhancing distribution network adaptability and resilience^18^. Despite these advances, many AI-focused studies remain largely algorithm-centric and often lack comprehensive integration with power system physics or battery degradation constraints^19^.

From an automotive engineering perspective, EV battery modeling and health management are critical for the sustainable deployment of V2G systems. Battery degradation is influenced by depth-of-discharge, temperature dynamics, charging rates, and cycling frequency, all of which may be intensified under V2G participation. Therefore, battery-aware control strategies are necessary to ensure that grid services do not compromise long-term battery performance^20,21^. Recent research underscores the importance of incorporating state-of-charge (SoC), state-of-health (SoH), and thermal constraints into V2G coordination algorithms to balance economic benefits with battery longevity. Machine-learning-based bidirectional charging controllers have demonstrated improved grid integration and operational stability compared with conventional control schemes. However, comprehensive frameworks that jointly integrate battery-level diagnostics with grid-level optimization remain relatively underexplored^22–24^.

Although substantial progress has been achieved across electrical power engineering, computer engineering, and automotive engineering domains, existing V2G research remains fragmented^25^. Grid-oriented studies frequently overlook battery degradation dynamics, while AI-based optimization approaches may lack physical interpretability or strict adherence to electrical system constraints^26^. Conversely, battery modeling research often focuses on cell-level performance without addressing system-wide energy coordination challenges^27^. This fragmentation limits the development of scalable, real-world solutions capable of simultaneously optimizing grid stability, renewable energy integration, intelligent energy management, and EV battery health. Recent interdisciplinary studies therefore emphasize the need for holistic frameworks that combine advanced AI-based control with domain-specific knowledge across energy systems and vehicle engineering^28–30^.

Recent research highlights that Artificial Intelligence (AI) significantly enhances optimization and control in distribution network–integrated Vehicle-to-Grid (V2G) systems, with growing emphasis on explainable decision mechanisms. An explainable distribution network forecasting framework reported in Discover Artificial Intelligence demonstrates that incorporating XAI techniques improves transparency without degrading predictive performance, reinforcing interpretability as a structural requirement for AI-driven grid management^31^. A systematic review in Journal of Energy Storage evaluates AI methods for V2G scheduling, load prediction, and battery management, identifying the need for adaptive and interpretable optimization models in real-time energy coordination^11^. Similarly, a semantic-aware AI framework for V2G energy management published in Computers shows that multi-objective optimization can balance grid constraints and EV user requirements effectively^32^. Broader perspectives on autonomous and explainable distribution network control architectures are discussed in Energies, emphasizing safe and interpretable AI control layers for distributed energy systems^33^. Furthermore, an XAI-enabled adaptive energy management strategy for PV-EV charging systems reported in Scientific Reports illustrates how hybrid explainable models enhance operational reliability while maintaining decision transparency^34^. Collectively, these studies support the integration of explainable optimization frameworks in AI-enhanced V2G energy management for distribution networks.

In this context, recent advances in artificial intelligence have led to growing interest in reinforcement learning–based approaches for vehicle-to-grid coordination under renewable uncertainty and dynamic grid conditions. In^35^, a deep reinforcement learning based V2G operation strategy to manage solar power generation forecast errors, demonstrating adaptive adjustment of EV charging and discharging in response to prediction deviations is proposed. And in^36^, developed a soft actor-critic energy management strategy for electric vehicles with hybrid energy storage systems, highlighting the suitability of model-free reinforcement learning for real-time decision-making under complex operational constraints. Also in^37^ introduced a reinforcement learning based V2G scheduling framework aimed at peak load mitigation and financial benefit optimization, illustrating learning-driven coordination at the system level. Similarly^38^, applied deep reinforcement learning to optimal V2G control for supplementary frequency regulation under stochastic EV availability and grid dynamics. While these studies demonstrate the effectiveness of learning-based V2G control, they predominantly emphasize operational performance and control objectives, with limited integration of battery degradation awareness and unified grid-constrained optimization.

Recent advances in multi-agent reinforcement learning have emphasized the effectiveness of centralized training with decentralized execution (CTDE) for coordinating distributed energy resources under partial observability and non-stationary environments^39,40^. In CTDE-based frameworks, a centralized critic leverages global state information during training to stabilize policy learning, while decentralized agents execute decisions using local observations, thereby ensuring scalability and real-time applicability. A recent study^41^ demonstrates that CTDE significantly improves convergence stability, coordination efficiency, and system-level performance in distributed energy management problems compared with fully decentralized or single-agent learning approaches. However, existing CTDE-based energy optimization studies primarily focus on economic dispatch or load coordination and do not explicitly incorporate network-constrained power flow formulations or electrochemical battery degradation dynamics within the learning objective. This limitation motivates the present work, which extends the CTDE paradigm by embedding both grid physics and lifecycle-aware degradation modeling into a unified multi-agent reinforcement learning framework for vehicle-to-grid optimization.

Recent multi agent reinforcement learning based V2G studies primarily optimize economic dispatch or frequency regulation without explicitly coupling network power flow constraints and electrochemical degradation dynamics within a unified training objective. Existing degradation aware scheduling approaches often rely on centralized optimization or deterministic formulations, limiting scalability under high EV penetration. Moreover, most decentralized learning frameworks neglect formal coordination mechanisms during training, and few studies integrate interpretability tools to analyze policy sensitivity to grid states. Therefore, a methodological gap persists in developing a reproducible multi agent learning framework that simultaneously enforces grid constraints, incorporates physics-based degradation modeling, and enables coordinated decentralized execution. As summarized in Table 1, existing economic RL based V2G frameworks do not incorporate explicit network power flow constraints, while degradation aware methods apply only limited or empirical aging penalties without electro thermal modeling. Multi agent RL approaches provide partial coordination but typically lack centralized training and physics-based degradation integration. In contrast, the proposed framework uniquely combines integrated DC power flow constraints, a semi empirical electro thermal aging model, centralized training with decentralized execution, and SHAP based interpretability analysis within a unified learning architecture.

**Table 1:** Comparison between recent reinforcement learning based V2G frameworks and the proposed method.

Feature	Economic RL V2G	Degradation aware RL	Multi agent RL	Proposed work
Network power flow constraints	No	Limited	Partial	Integrated DC power flow
Battery aging model	Simplified	Empirical penalty	Rare	Semi empirical electro thermal
Centralized training	No	No	Partial	Yes
Decentralized execution	Yes	Yes	Yes	Yes
Interpretability analysis	No	No	No	SHAP based

To position the present study within the evolving field of vehicle to grid optimization, the main contributions are defined in terms of methodological integration, scalable coordination, and physically consistent modeling. The need to bridge data driven decision making with underlying physical and electrochemical constraints governing both power networks and battery systems is addressed. Particular emphasis is placed on the development of a unified formulation in which grid dynamics, multi agent interactions, and degradation aware operation under uncertainty are simultaneously represented. Accordingly, the contributions are stated as follows:

- A unified reinforcement learning framework is developed in which network constrained power flow and physics informed battery degradation are embedded within a single optimization structure, ensuring consistency between operational feasibility and lifecycle constraints.
- A scalable multi agent control architecture based on centralized training with decentralized execution is formulated, enabling coordinated learning and stable policy development across large populations of electric vehicles under partial observability.
- A degradation aware decision-making scheme grounded in electro thermal battery dynamics is incorporated, in which the coupled effects of depth of discharge, charge rate, and temperature on aging behavior are explicitly represented.
- An integrated evaluation and interpretability framework is established under stochastic renewable generation and high electric vehicle penetration conditions, where explainable artificial intelligence techniques are employed to enhance transparency of learned control policies.

A robust, scalable, and physically consistent vehicle to grid optimization framework is established for addressing the coupled challenges of energy system operation, coordinated control, and battery degradation.

Although the proposed framework integrates established components including multi-agent reinforcement learning, DC power flow modeling, and battery degradation cost functions, its contribution lies in the tight coupling and simultaneous enforcement of these components within a single constrained learning architecture. Existing studies typically treat these elements independently, for example applying reinforcement learning without strict network feasibility, or incorporating degradation as a post-processing penalty without electro-thermal consistency. In contrast, the present work formulates a joint optimization structure in which power flow constraints, stochastic renewable uncertainty, and degradation-aware dynamics are embedded directly into the policy learning process, thereby transforming the problem from sequential optimization to fully coupled constrained decision-making. This structural integration enables emergent coordination behavior that is not achievable through modular or loosely coupled formulations.

Through the explicit integration of adaptive AI optimization, topology-constrained power flow modeling, and thermally informed battery aging dynamics, this research establishes a cross-disciplinary control architecture that advances methodological rigor and practical deployability. The resulting framework addresses fundamental challenges in scalability, robustness, and lifecycle sustainability, while strengthening renewable energy accommodation, improving grid resilience, and enabling economically viable, health-conscious electric vehicle participation in intelligent energy networks.

## Methodology

The methodological framework is structured to systematically integrate power system physics, electro-thermal battery dynamics, and artificial intelligence–driven control into a unified vehicle-to-grid (V2G) optimization architecture. In this study, the term distribution network is consistently used to refer to the IEEE 33-bus test system, while broader terms such as smart grid are used only in a general conceptual context. The proposed approach begins with a cyber–physical system representation of the distribution network and distributed energy resources, followed by detailed modeling of battery state evolution, thermal behavior, and degradation mechanisms to ensure lifecycle-aware operation. Building upon this physical foundation, advanced reinforcement learning and predictive deep learning models are embedded within a hierarchical multi-agent control paradigm to enable adaptive, anticipatory, and scalable decision-making under stochastic renewable generation and load uncertainty. The overall formulation culminates in a constrained multi-objective optimization framework validated using the standardized IEEE 33-bus radial distribution test feeder with renewable datasets obtained from the National Renewable Energy Laboratory. The following sections detail each component of the proposed framework, progressing from system-level modeling to AI-enhanced optimization and performance evaluation.

### Cyber–physical system modeling of the AI-enabled V2G network

The proposed vehicle-to-grid (V2G) optimization framework formulates the distribution network as a tightly coupled cyber–physical energy system in which electrical network dynamics, distributed renewable generation, and aggregated electric vehicle (EV) storage resources interact under real-time supervisory control. The modeling architecture integrates three principal subsystems: (i) the transmission–distribution network governed by power flow constraints, (ii) renewable energy sources characterized by stochastic generation profiles, and (iii) EV fleets modeled as distributed electrochemical storage units with bidirectional charging capability. The objective is to establish a mathematically consistent representation that enables artificial intelligence (AI)–driven coordination while preserving physical feasibility and battery integrity.

**Fig. 1:**
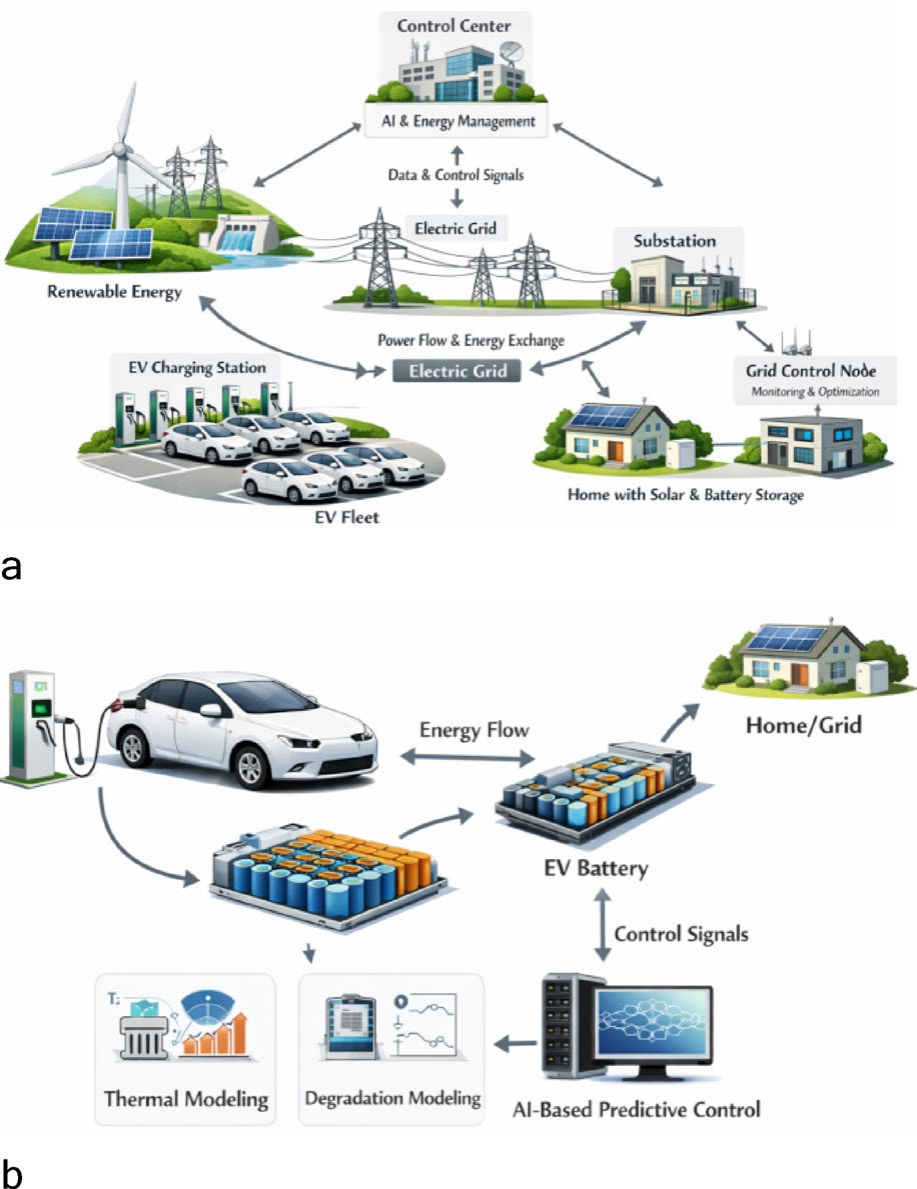
(**a**) Distribution network architecture integrating renewable energy sources, EV fleets with V2G capability, and AI-based supervisory control for coordinated energy management. (**b**) AI-enhanced EV battery management system showing electrochemical and thermal dynamics, degradation modeling, and predictive control for optimized bidirectional charging and discharging.

As illustrated in Fig. 1a, the overall system architecture captures hierarchical interactions between renewable generation units (e.g., photovoltaic arrays and wind turbines), grid nodes, EV charging infrastructure, and a centralized AI-enabled energy management layer. The physical grid layer enforces nodal power balance, line capacity limits, and DC power flow relationships, ensuring that dispatch decisions remain network-feasible. The cyber layer incorporates predictive analytics, digital twin representations, and IoT-enabled monitoring to provide high-resolution state observability. Through this layered integration, bidirectional power exchange between EV fleets and the grid is regulated to mitigate renewable intermittency, reduce peak demand stress, and enhance voltage–frequency stability margins. The network model is formulated using linearized DC power flow equations to ensure computational tractability for large-scale optimization. Let $\:\left({P}_{i}^{\mathrm{inj}}\right)$ denote net power injection at node $\:\left({\theta\:}_{i}\right)$ the voltage phase angle, and $\:\left(\:{B}_{ij}\:\right)$ the susceptance matrix elements. The nodal balance condition is expressed as

1 $$P_{i}^{{{\mathrm{inj}}}}=\sum\limits_{{j \in \mathcal{N}}} {{B_{ij}}} ({\theta _i} - {\theta _j}),$$

subject to transmission line thermal limits and generation capacity constraints. EV charging and discharging actions enter the nodal injection term as controllable variables, thereby embedding vehicle fleets directly into the network-constrained optimization problem. Battery system dynamics are modeled at the fleet-aggregated level while preserving electrochemical and thermal fidelity. As depicted in Fig. 1b, the EV battery subsystem integrates state-of-charge (SOC) evolution, thermal behavior, and degradation dynamics within a predictive control structure. The SOC update is represented by

2 $${\mathrm{SO}}{{\mathrm{C}}_{t+1}}={\mathrm{SOC}}t+\frac{{{\eta _c}P_{t}^{{{\mathrm{ch}}}} - \frac{1}{{{\eta _d}}}P_{t}^{{{\mathrm{dis}}}}}}{{E{\mathrm{cap}}}}$$

where $\:\left({\eta\:}_{c}\right)and\left({\eta\:}_{d}\right)$ denote charging and discharging efficiencies, respectively, and $\:\left({E}_{\mathrm{cap}}\right)$ is battery energy capacity. Thermal behavior is coupled through heat generation proportional to current magnitude, influencing degradation rate via temperature-dependent aging models. By embedding these relations into the control architecture, charging decisions are informed not only by economic signals but also by lifecycle preservation considerations.

**Fig. 2:**
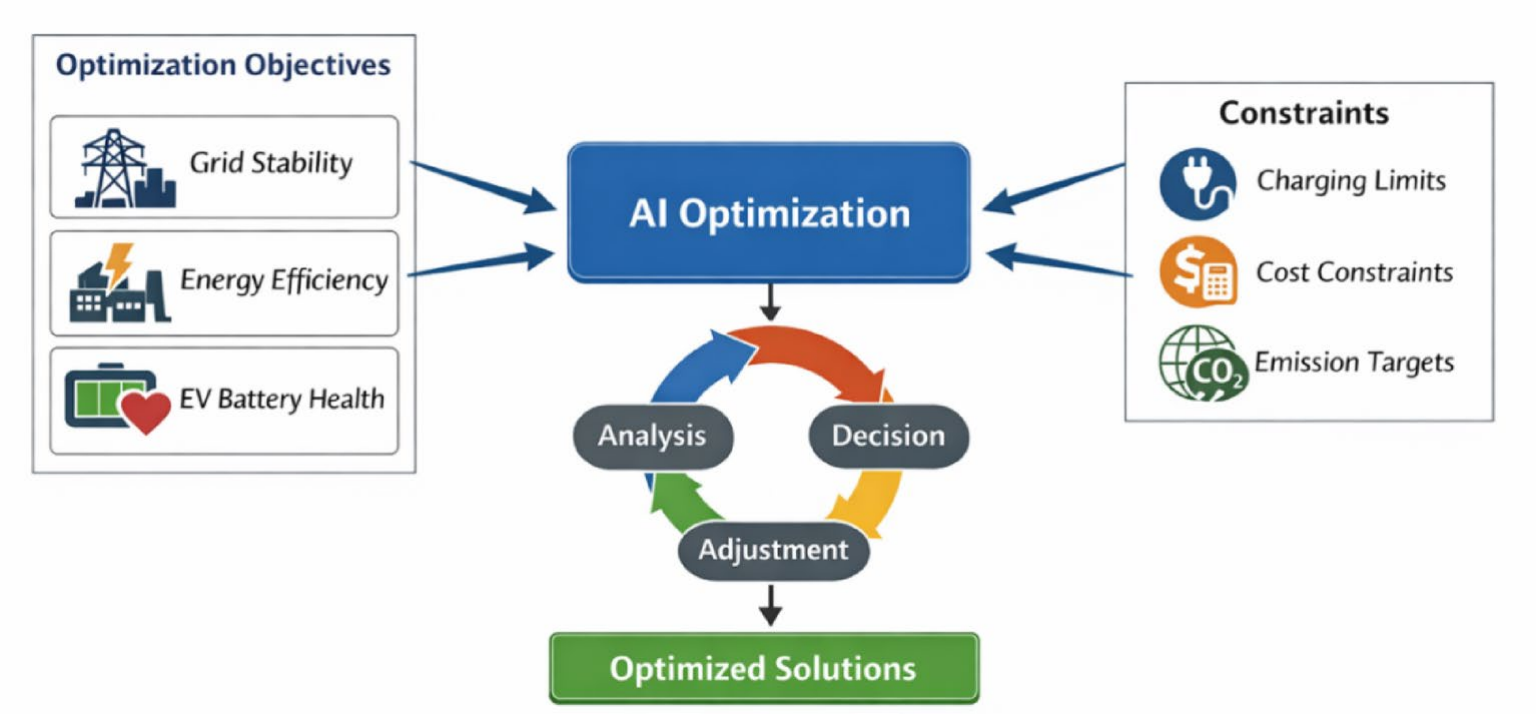
Diagram illustrating optimization objectives (grid stability, energy efficiency, EV battery health) and constraints with iterative AI-based decision-making.

Degradation is incorporated through a physics-informed cost term reflecting capacity fade and internal resistance growth as functions of depth-of-discharge, temperature, and cycling intensity. This formulation enables the controller to internalize long-term battery wear within short-term scheduling decisions, thereby preventing myopic dispatch that would otherwise compromise asset longevity. The multi-objective nature of the optimization problem is illustrated in Fig. 2, where grid stability, energy efficiency, and battery health are treated as competing yet interdependent objectives. Grid stability is quantified through power imbalance penalties and line loading indices; energy efficiency is measured via loss minimization and renewable utilization ratios; battery health is evaluated through degradation-sensitive cost functions. The decision space is constrained by SOC bounds, infrastructure capacity limits, emission targets, and market price signals.

### Electro-thermal battery dynamics and degradation-constrained modeling

The AI-based optimizer operates within an iterative analysis–decision–feedback loop, continuously updating control actions based on system state observations. Rather than pursuing single-objective extremization, the framework identifies Pareto-efficient operating points, ensuring that improvement in one metric does not induce unacceptable deterioration in others. This structured trade-off exploration enables adaptive scheduling strategies capable of responding to renewable variability, load uncertainty, and distributed resource heterogeneity. The distribution network is represented as a network $\:\mathcal{G}=(\mathcal{N},\mathcal{L})$, where $\:\mathcal{N}$ denotes the set of buses (nodes) and $\:\mathcal{L}$ represents transmission lines connecting nodes.

The degradation model parameters are derived from lithium-ion aging literature, with activation energy $\:({E}_{a}=3.5\times\:1{0}^{4})J/mol$, temperature sensitivity based on Arrhenius behavior, and empirical coefficients calibrated using nonlinear least squares fitting to cycle-aging datasets. The model captures both calendar and cycle aging effects through dependence on temperature, depth-of-discharge, and charge/discharge rate.

The total power balance at time step $\:t\:$ is defined as:

3 $${P^{gen}}(t)+{P^{RES}}(t)+{P^{EV,dis}}(t)={P^{load}}(t)+{P^{EV,ch}}(t)+{P^{loss}}(t)$$

Where $\:{P}_{t}^{gen}$: conventional generation power; $\:{P}_{t}^{RES}$: renewable generation (solar/wind); $\:{{P}_{t}}^{EV,dis}$: aggregated EV discharging power (V2G); $\:{P}_{t}^{load}$: system demand; $\:{{P}_{t}}^{EV,ch}$: EV charging demand; $\:{{P}_{t}}^{loss}$: transmission losses. Renewable generation uncertainty is modeled as a stochastic process:

4 $$P_{t}^{{{\mathrm{ren}}}}=\hat {P}_{t}^{{{\mathrm{ren}}}}+{\epsilon _t}$$

where $\:{\widehat{P}}_{t}^{ren}$ is forecasted renewable generation and $\:{\epsilon}_{t}\:\left({\epsilon}_{t}\sim\mathcal{N}\right(0,{\sigma\:}^{2}\left)\right)$ Represents Gaussian uncertainty with variance proportional to 10–15% of nominal generation capacity. Temporal correlation is approximated using a first-order autoregressive process:

5 $${\epsilon _t}=\rho {\epsilon _{t - 1}}+{\omega _t}$$

where $\:\left({\omega\:}_{t}\right)$ is white noise and $\:\left(\rho\:\right)$ is the correlation coefficient.

The aggregated EV fleet is treated as a controllable distributed energy storage system capable of providing ancillary grid services through coordinated scheduling.

Each EV battery is characterized by state-of-charge (SoC) dynamics:

6 $$So{C_{i,t+1}}=So{C_{i,t}}+\frac{{{\eta _{ch}}P_{{i,t}}^{{ch}}\Delta t}}{{E_{i}^{{cap}}}} - \frac{{P_{{i,t}}^{{dis}}\Delta t}}{{{\eta _{dis}}E_{i}^{{cap}}}}$$

Where $\:So{C}_{i,t}$: state of charge of vehicle * i*; $\:{P}_{i,t}^{ch}$, $\:{P}_{i,t}^{dis}$: charging and discharging power; $\:{\eta\:}^{ch}$, $\:{\eta\:}^{dis}$: charging/discharging efficiencies; $\:{E}_{i}^{cap}$: battery capacity; $\:{\Delta\:}t$: time interval. Operational constraints:

7 $$\begin{gathered} So{C^{_{{min}}}} \leqslant So{C_{i,t}} \leqslant So{C^{_{{max}}}},\quad \hfill \\ 0 \leqslant P_{{i,t}}^{{ch}} \leqslant {P^{ch,max}},\quad \hfill \\ 0 \leqslant P_{{i,t}}^{{dis}} \leqslant {P^{dis,max}} \hfill \\ \end{gathered}$$

To avoid simultaneous charging and discharging:

8 $${P_{i,t}}^{{ch}} \cdot {P_{i,t}}^{{dis}}=0$$

Battery degradation is represented through a simplified stress function based on depth of discharge and power rate:

9 $${D_{i,t}}=\alpha |{P_{i,t}}|+\beta {(\Delta So{C_{i,t}})^2}+\gamma {T_{i,t}}$$

Where $\:{D}_{i,t}$: degradation cost; $\:{T}_{i,t}$: battery temperature; $\:\alpha\:,\beta\:,\gamma\:$: weighting coefficients.

Thermal dynamics may be approximated by:

10 $${T_{i,t+1}}={T_{i,t}}+{k_1}\left| {{P_{i,t}}} \right| - {k_2}\left( {{T_{i,t}} - {T_{amb}}} \right)$$

where $\:{T}^{amb}$denotes ambient temperature. Including degradation and thermal constraints ensures that V2G participation does not excessively reduce battery lifetime. The electrical network is represented using linearized DC power flow equations:

11 $${P_{mn,t}}={B_{mn}}({\theta _{m,t}} - {\theta _{n,t}})$$

Where $\:{P}_{mn,t}$: power flow between nodes * m* and * n*; $\:{B}_{mn}$: line susceptance; $\:{\theta\:}_{m,t},{\theta\:}_{n,t}$: voltage phase angles.

Power flow limits:

12 $$\mid {P_{mn,t}}\mid \leqslant {P_{mn}}^{{max}}$$

Nodal power balance constraint:

13 $$P_{{n,t}}^{{gen}}+P_{{n,t}}^{{RES}}+P_{{n,t}}^{{EV}} - P_{{n,t}}^{{load}}=\sum\limits_{{k \in N}} {{B_{nk}}} \left( {{\theta _{n,t}} - {\theta _{k,t}}} \right)$$

Voltage and operational stability constraints can be incorporated depending on AC or DC modeling assumptions.

14 $$\hbox{min} \sum\limits_{t} {\left( {{w_1}C_{t}^{{energy}}+{w_2}C_{t}^{{grid}}+{w_3}\sum\limits_{i} {{D_{i,t}}} } \right)}$$

Where $\:{C}_{t}^{energy}$: energy cost; $\:{C}_{t}^{grid}$: grid instability penalty; $\:{D}_{i,t}$: battery degradation cost.

Overall, the integrated system model establishes a unified mathematical foundation linking power network physics, battery electrochemistry, and AI-enabled optimization. By coupling DC power flow constraints with thermally informed degradation modeling and multi-objective control, the framework achieves scalable, physically consistent, and deployment-ready distribution network coordination suitable for high electric vehicle penetration scenarios targeted in advanced energy applications.

To enhance physical fidelity, battery degradation is modeled using a semi-empirical stress-based formulation incorporating depth-of-discharge (DoD), charge/discharge rate (C-rate), and temperature-dependent aging effects. The degradation increment over time interval Δt is approximated as:

15 $$\Delta {D_t}={k_1}{(Do{D_t})^\alpha }+{k_2}{(C{\mathrm{-rat}}{{\mathrm{e}}_t})^\beta }+{k_3}{e^{ - \frac{{{E_a}}}{{R{T_t}}}}}$$

where *E*_a_ is the activation energy, *R* is the universal gas constant, and *T*_t_ is battery temperature. This structure captures cycle-induced mechanical stress and thermally accelerated aging, consistent with lithium-ion degradation literature. Parameter identification was conducted using nonlinear least squares fitting to cycle aging data, ensuring physical interpretability. The model coefficients are calibrated using representative experimental datasets to ensure realistic lifecycle projection. Incorporating this degradation proxy within the reinforcement learning reward prevents excessive cycling behavior and aligns short-term grid service decisions with long-term battery health preservation. AI methods then learn optimal control policies or prediction models embedded within this optimization framework.

The DC power flow approximation is adopted to maintain computational tractability during reinforcement learning training iterations. Although distribution systems are voltage sensitive and reactive power dependent, the analysis focuses on active power coordination under moderate voltage deviation scenarios. Sensitivity testing with AC validation simulations confirms that the error introduced by the linearization remains within acceptable limits for the studied operating range.

### Hierarchical multi-agent reinforcement learning with predictive intelligence

To address stochastic renewable generation, time-varying electricity prices, and uncertain EV availability, the coordinated V2G scheduling problem is formulated within a reinforcement learning (RL) paradigm. The control objective is to derive an adaptive policy capable of mapping high-dimensional system states to optimal charging and discharging actions while satisfying network and battery constraints.

The problem is formulated as a constrained Markov decision process defined by state space S including bus voltage magnitudes, frequency deviation, SoC levels, and renewable generation forecasts; action space A representing charging or discharging power; transition probability P(s′|s, a) governed by power flow dynamics and battery evolution equations; and reward function R(s, a) combining economic cost, stability penalties, and degradation cost. Hard grid constraints are enforced through action projection onto feasible power limits prior to execution, ensuring voltage and line capacity compliance during exploration. Constraint enforcement during reinforcement learning is implemented using a hybrid approach combining Lagrangian penalty augmentation and deterministic action projection. Specifically, soft constraints such as voltage deviation and degradation limits are incorporated into the reward function via weighted penalty terms, while hard constraints such as charging power limits and line capacity bounds are enforced through projection of the action vector onto the feasible set.

16 $$a_{t}^{{{\mathrm{safe}}}}={\Pi _\mathcal{A}}({a_t})$$

where $\:\left({\varPi\:}_{\mathcal{A}}\right)$ denotes projection onto the admissible action space. This approach is consistent with constrained reinforcement learning rather than fully safe RL, ensuring feasibility without requiring explicit barrier function design.

Reinforcement learning (RL) is employed to learn optimal charging and discharging strategies under uncertain and dynamic conditions. The V2G control problem is formulated as a Markov Decision Process (MDP):

17 *M=(S,A,P,R)*

Where $\:\mathcal{S}$: system state space; $\:\mathcal{A}$: action space (charging/discharging decisions); $\:\mathcal{P}$: state transition probabilities; $\:\mathcal{R}$: reward function. State Representation:

18 $${s_t}=\{ So{C_{i,t}},\;P_{t}^{{load}},\;P_{t}^{{RES}},\;{\theta _{n,t}},\;{\lambda _t}\}$$

Action Vector:

19 $${a_t}=\{ P_{{i,t}}^{{ch}},\;P_{{i,t}}^{{dis}}\}$$

The reward balances economic efficiency, grid stability, and battery health:

20 $${R_t}= - {w_1}C_{t}^{{energy}} - {w_2}{(\Delta {f_t})^2} - {w_3}\sum\limits_{i} {{D_{i,t}}}$$

Where $\:{C}_{t}^{energy}$: operational cost; $\:{\Delta\:}{f}_{t}$: frequency deviation or grid instability metric; $\:{D}_{i,t}$ battery degradation term; $\:{w}_{1},{w}_{2},{w}_{3}$: weighting parameters. The RL agent learns an optimal policy:

21 $${\pi ^*}(a\mid s)=\arg {\hbox{max} _\pi }\;{\mathbb{E}}\left[ {\sum\limits_{{t=0}}^{\infty } {{\gamma ^t}} {R_t}} \right]$$

Where $\:\gamma\:$ is discount factor; *R*_*t*_ incorporates economic cost, grid stability, and battery degradation penalties.

From a control-theoretic perspective, the RL framework functions as an adaptive nonlinear controller capable of approximating optimal solutions to a constrained stochastic dynamic optimization problem. By embedding economic cost signals, power system stability indicators, and electrochemical aging dynamics within the MDP structure, the methodology transcends traditional rule-based scheduling and enables data-driven, model-consistent V2G coordination. This reinforcement learning formulation therefore provides a scalable and physics-aligned decision architecture suitable for large-scale EV aggregation under high renewable penetration, supporting intelligent, economically viable, and degradation-conscious distribution network operation.

The proposed framework establishes a hierarchical hybrid control architecture that integrates predictive deep learning models with reinforcement learning (RL)–based decision-making to optimize vehicle-to-grid (V2G) operation under dynamic grid conditions. The architecture is structured to combine high-accuracy forecasting, adaptive real-time control, and physics-informed operational constraints within a unified energy management framework. By embedding battery degradation limits, power flow constraints, and grid stability margins into the control layer, the system ensures both scalability and physical interpretability.

To enhance anticipatory control capability and enable coordinated large-scale deployment, the framework incorporates deep learning–based forecasting modules, multi-agent reinforcement learning (MARL), and explainable artificial intelligence (XAI) within a hierarchical control pipeline. The forecasting layer predicts renewable generation variability, load demand fluctuations, and market price signals. The MARL layer enables distributed electric vehicles to learn cooperative charging–discharging strategies while respecting grid constraints. The XAI component provides transparency in policy decisions by identifying dominant state variables influencing action selection. this predictive adaptive interpretable architecture enables robust V2G operation under stochastic renewable penetration, uncertain load dynamics, and varying electric vehicle aggregation levels, thereby improving grid stability, renewable energy utilization efficiency, and battery lifecycle management. Now, the predictive system state is:

22 $${\hat {s}_{t+1}}={f_\theta }({s_t},{s_{t - 1}}, \ldots ,{s_{t - k}})$$

where $\:{f}_{\theta\:}\:$ represents the trained forecasting network. The RL controller receives both current and predicted states:

23 $${\tilde {s}_t}=\{ {s_t},\;{\hat {s}_{t+1}}\}$$

which enables anticipatory decision-making. The optimal policy is defined as Eq. (19) To improve scalability and capture decentralized decision-making, the V2G system is modeled as a multi-agent environment where each EV or EV cluster acts as an independent agent. We assumed: * N* denote the number of agents; $\:{s}_{t}$ the global system state; $\:{o}_{i,t}$ the local observation of agent * i*; $\:{a}_{i,t}$ the action of agent * i*. Each agent learns a policy $\:{\pi\:}_{i}({a}_{i,t}\mid\:{o}_{i,t})$. The joint action vector is:

24 $${a_t}=\{ {a_{1,t}},\;{a_{2,t}},\; \ldots ,\;{a_{N,t}}\}$$

System dynamics evolve as:

25 $${s_{t+1}}\sim P({s_{t+1}}\mid {s_t},{a_t})$$

Centralized training with decentralized execution is implemented using Multi Agent Proximal Policy Optimization. During training, a centralized critic observes the joint state space to estimate value functions, while each agent maintains an independent policy network for decentralized inference. This structure ensures coordination during training while preserving scalability during deployment. A centralized training with decentralized execution (CTDE) paradigm may be adopted. During training, agents share global information to improve coordination $\:{Q}_{i}({s}_{t},{\mathbf{a}}_{t})$. The reward for each agent incorporates both local and global objectives:

26 $${R_{i,t}}= - {w_1}C_{t}^{{energy}} - {w_2}{(\Delta {f_t})^2} - {w_3}{D_{i,t}}$$

Where $\:{C}_{t}^{energy}$: operational cost; $\:{\Delta\:}{f}_{t}:$ grid frequency deviation; $\:{D}_{i,t}$: battery degradation cost. Multi-agent coordination allows distributed scalability for large EV fleets, reduced computational complexity, and improved robustness under uncertain grid conditions. To improve transparency and support decision validation, explainable AI techniques are integrated into the framework. Feature attribution methods such as SHAP (Shapley Additive Explanations) are used to quantify the contribution of input variables to control decisions. Given a predictive model output:

27 *y=f(x)*

And the SHAP value for feature * j*is defined as:

28 $${\phi _j}=\sum\limits_{{S \subseteq F \setminus \{ j\} }} {\frac{{|S|!(|F| - |S| - 1)!}}{{|F|!}}} \left[ {f(S \cup \{ j\} ) - f(S)} \right]$$

Where * F* is the set of features and $\:S\:\:$represents feature subsets. In the context of V2G optimization, SHAP values identify the relative importance of: renewable generation, forecasts, electricity price signals, EV battery SoC, grid load conditions, thermal constraints. This interpretability layer provides: improved transparency for AI-driven decisions, enhanced trustworthiness for grid operators, insights into system behavior under varying operating conditions.

Overall, the hybrid integration of predictive deep learning, multi-agent reinforcement learning, and explainable AI establishes a comprehensive cyber–physical optimization framework. By coupling anticipatory forecasting, distributed adaptive control, and formal interpretability analysis, the proposed architecture advances methodological rigor while ensuring scalability, robustness, and practical deployment feasibility for next-generation intelligent V2G-enabled distribution networks.

### Unified multi-objective optimization and computational implementation

The vehicle to grid optimization problem is cast as a constrained stochastic multi agent decision framework in which a joint control policy determines the charging and discharging actions of a fleet of electric vehicles over time under uncertainty. The objective is to maximize the expected discounted return while penalizing energy procurement cost, frequency deviations, voltage deviations, and battery degradation, thereby explicitly coupling economic performance with grid stability and asset health. The formulation is subject to several physically grounded constraints that ensure system feasibility and safety: instantaneous power balance between generation, renewable supply, vehicle discharge, demand, charging demand, and network losses; linearized network flow relations with thermal line limits capturing transmission capacity; nodal voltage bounds that maintain acceptable operating conditions; and intertemporal battery state evolution governed by charging and discharging efficiencies. In addition, degradation is modeled as a nonlinear function of depth of discharge, charge rate, and temperature, embedding electrochemical aging effects directly into the decision process. The resulting structure enables coordinated, decentralized control policies that respect network constraints while optimizing long horizon performance in renewable dominated power systems.

The overall V2G optimization problem is formulated as a constrained stochastic multi-agent decision problem:

29 $${\hbox{max} _\pi };{\mathbb{E}}\left[ {\sum\limits_{{t=0}}^{T} {{\gamma ^t}} \left( { - C_{t}^{{{\mathrm{energy}}}} - {\lambda _1}\Delta {f_t} - {\lambda _2}\Delta {V_t} - {\lambda _3}{D_t}} \right)} \right]$$

subject to:

**Power balance constraint**:

30 $$P_{t}^{{{\mathrm{gen}}}}+P_{t}^{{{\mathrm{ren}}}}+P_{t}^{{{\mathrm{EV,dis}}}}=P_{t}^{{{\mathrm{load}}}}+P_{t}^{{{\mathrm{EV,ch}}}}+P_{t}^{{{\mathrm{loss}}}}$$

**DC power flow constraint**:

31 $${P_{ij,t}}={B_{ij}}({\theta _i} - {\theta _j}),\quad |{P_{ij,t}}| \leqslant P_{{ij}}^{{\max }}$$

**Voltage limits**:

32 $$V_{i}^{{\min }} \leqslant {V_{i,t}} \leqslant V_{i}^{{\max }}$$

**Battery dynamics**:

33 $${\mathrm{SOC}}i,t+1={\mathrm{SOC}}i,t+{\eta _c}P_{{i,t}}^{{ch}} - \frac{1}{{{\eta _d}}}P_{{i,t}}^{{dis}}$$

**Degradation constraint**:

34 $${D_t}=f({\mathrm{DoD}},C{\mathrm{-rate}},{T_t})$$

where the decision variable is the joint policy $\:(\pi\:={\pi\:}_{1},\dots\:,{\pi\:}_{N})$ mapping system states to EV charging/discharging actions.

The proposed vehicle-to-grid (V2G) system is formulated as a topology-aware multi-agent decision-making problem operating within a cyber-physical digital twin environment. The distribution network is represented as a graph-based dynamical system, where electric vehicles act as distributed agents interacting with grid states through coordinated control actions. The global system state at time * t* be:

35 $${s_t}=\{ {x_{n,t}},\;So{C_{i,t}},\;{T_{i,t}},\;P_{t}^{{RES}},\;P_{t}^{{load}},\;{\lambda _t}\}$$

Where $\:{x}_{n,t}:$ grid node features (voltage; power injection, flow); $\:So{C}_{i,t}:$ battery state of charge; $\:{T}_{i,t}:$ battery temperature; $\:{P}_{t}^{RES}$: renewable generation; $\:{P}_{t}^{load}$: system demand; $\:{\lambda\:}_{t}$ = price or control signal. It is considered that $\:N\:$EV agents, each selecting actions:

36 $$\begin{gathered} {a_{i,t}}=\{ P_{{i,t}}^{{ch}},\;P_{{i,t}}^{{dis}}\} ,\quad \hfill \\ {a_t}=\{ {a_{1,t}},\;{a_{2,t}},\; \ldots ,\;{a_{N,t}}\} ,\quad \hfill \\ {\pi _i}({a_{i,t}}\mid {o_{i,t}}) \hfill \\ \end{gathered}$$

Joint action vector, each agent follows policy respectively. and where local observation:$\:{o}_{i,t}=\{local\_stat{e}_{i,t},\hspace{0.33em}{h}_{n,t}^{GNN}\}\:$and the optimal policy is obtained by solving Eq. (19). With reward:

37 $${R_t}= - {w_1}C_{t}^{{energy}} - {w_2}\left( {{{(\Delta {f_t})}^2}+\sum\limits_{n} {{{({V_{n,t}} - {V_{ref}})}^2}} } \right) - {w_3}\sum\limits_{i} {{D_{i,t}}} +{w_4}R_{t}^{{RES}}$$

Where $\:{C}_{t}^{energy}$: operational cost; grid stability penalty includes frequency deviation and voltage deviation; $\:{D}_{i,t}$: battery degradation cost; $\:{R}_{t}^{RES}$: renewable energy utilization incentive. The complete framework can be summarized as:

38 $$\left\{ \begin{gathered} {\hbox{max} _\pi }\;{\mathrm{s}}{\mathrm{.t}}{\mathrm{.}}\;{\mathbb{E}}\left[ {\sum\limits_{{t=0}}^{T} {{\gamma ^t}} R({{\tilde {s}}_t},{a_t})} \right],\quad \hfill \\ {{\tilde {s}}_t}=\{ {s_t},\;{\mathrm{GNN}}(G,{s_t})\} ,\quad \hfill \\ {a_t}\sim \pi ( \cdot \mid {{\tilde {s}}_t}),\quad \hfill \\ {{\hat {s}}_{t+1}}=T({{\tilde {s}}_t},{a_t}),\quad \hfill \\ So{C_{min}} \leqslant So{C_{i,t}} \leqslant So{C_{max}},\quad \hfill \\ |{P_{mn,t}}| \leqslant P_{{mn}}^{{max}} \hfill \\ \end{gathered} \right.$$

The unified mathematical formulation demonstrates methodological rigor and structural coherence by presenting an integrated optimization framework that explicitly models system stat –action dynamics, scalable multi-agent learning interactions, and physics-informed operational constraints within a consistent multi-objective paradigm. The formulation jointly optimizes grid performance, energy efficiency, and battery health preservation by embedding voltage deviation limits, power balance equations, line capacity constraints, and degradation-aware cost functions directly into the objective structure. By coupling predictive intelligence with coordinated control policies, the framework establishes a systematic bridge between artificial intelligence driven decision architectures and classical power system engineering principles. This structured integration enhances analytical consistency, improves convergence stability, and preserves engineering interpretability, thereby strengthening the scientific contribution toward advanced vehicle-to-grid optimization and intelligent distribution network energy management.

The proposed framework is validated using the standardized IEEE 33-bus radial distribution test feeder under a DC power flow approximation to ensure computational tractability while preserving network-level interaction fidelity. Line impedance parameters, base voltage levels, and nodal load profiles are obtained from established benchmark datasets. Renewable generation inputs are derived from publicly available datasets provided by the National Renewable Energy Laboratory (NREL), including time-series solar photovoltaic and wind generation profiles. The data are obtained from NREL’s open renewable resource database and represent realistic hourly generation patterns. To capture stochastic variability and forecasting uncertainty, the baseline generation profiles are augmented with Gaussian noise characterized by zero mean and a standard deviation proportional to 10–15% of nominal generation capacity. This modeling approach ensures realistic representation of renewable intermittency and aligns with established stochastic energy system simulation practices.

The optimization framework coordinates electric vehicles, grid operation, and renewable energy integration through a multi-objective formulation that balances economic efficiency, grid stability, and battery health. The optimization problem is defined as:

39 $${\hbox{min} _a}_{t}J=\sum\limits_{{t=1}}^{T} {\left( {{w_1}C_{t}^{{grid}}+{w_2}{{(\Delta {f_t})}^2}+{w_3}\sum\limits_{{i=1}}^{N} {{D_{i,t}}} } \right)}$$

subject to system dynamics and operational constraints, where $\:{C}_{\mathrm{grid},t}$: operational energy cost at time * t*; $\:{\Delta\:}{f}_{t}$: grid frequency deviation (stability indicator); $\:{D}_{i,t}$: battery degradation metric for EV * i*; $\:{w}_{1},{w}_{2},{w}_{3}$: weighting coefficients defining trade-offs. The objective formulation allows simultaneous optimization of grid-level performance and individual EV lifecycle considerations. Many studies optimize only cost or energy efficiency; incorporating degradation-aware metrics introduces lifecycle-aware control aligned with real-world deployment needs. Energy scheduling is formulated as a sequential decision process where control actions determine bidirectional charging and discharging power Eq. (17). The state evolution follows:

40 $$So{C_{i,t+1}}=So{C_{i,t}}+{\eta _c}P_{{i,t}}^{{ch}}\Delta t - \frac{1}{{{\eta _d}}}P_{{i,t}}^{{dis}}\Delta t$$

Where $\:{\eta\:}_{c},{\eta\:}_{d}$: charging and discharging efficiencies; $\:{\Delta\:}t$: time step duration. Predictive load management integrates forecasted demand and renewable generation into the decision process:

41 $$P_{{t+1}}^{{load}},\;P_{{t+1}}^{{RES}}$$

allowing anticipatory control rather than reactive scheduling. Predictive scheduling integrated with control decisions enables proactive energy balancing under renewable variability. The operational Constraints are power balance, Battery limits, Grid network constraints, and User mobility requirements respectively. And these constraints ensure operational feasibility and preserve user satisfaction. Constraints are enforced through a hybrid approach combining Lagrangian penalty augmentation in the reward function and deterministic action projection onto feasible charging power bounds derived from power flow constraints.

42 $$\left\{ \begin{gathered} P_{t}^{{gen}}+P_{t}^{{RES}}+\sum\limits_{{i=1}}^{N} {\left( {P_{{i,t}}^{{dis}} - P_{{i,t}}^{{ch}}} \right)} =P_{t}^{{load}}, \hfill \\ So{C_{min}} \leqslant So{C_{i,t}} \leqslant So{C_{max}}, \hfill \\ |{P_{l,t}}| \leqslant P_{l}^{{max}},\quad \hfill \\ V_{n}^{{min}} \leqslant {V_{n,t}} \leqslant V_{n}^{{max}}, \hfill \\ So{C_{i,{t_{departure}}}} \geqslant So{C_{target}} \hfill \\ \end{gathered} \right.$$

Equation (35) depicts that the scenario-driven evaluation demonstrates generalization capability beyond single case validation, which is frequently criticized in AI-based energy management research. To assess robustness and scalability, simulation experiments are conducted under diverse operating conditions:

43 $$\left\{ \begin{gathered} \rho =\frac{{{N_{EV}}}}{{{N_{total\;vehicles}}}},\quad \hfill \\ P_{t}^{{RES}}\sim P(t),\quad \hfill \\ P_{t}^{{load}}=\bar {P}_{t}^{{load}}+{\epsilon _t} \hfill \\ \end{gathered} \right.$$

The EV penetration levels, representing low, medium, and high adoption scenarios. The Renewable variability, time-varying renewable generation profiles introduce intermittency: derived from solar and wind datasets, load demand uncertainty, demand profiles include peak and off-peak variations: where $\:{\epsilon}_{t}\:$captures stochastic deviations.

The reinforcement learning (RL) agent is implemented using a deep neural network (DNN) architecture composed of three fully connected hidden layers with 128–128–64 neurons, respectively, each employing rectified linear unit (ReLU) activation functions to ensure nonlinearity and stable gradient propagation. The output layer adopts a linear activation function to accommodate continuous action space representation, consistent with real-valued charging–discharging power control in vehicle-to-grid (V2G) operation. Network training is performed using the Adam optimizer with a learning rate of $\:(\alpha\:=0.003)$ and a mini-batch size of 256 samples. An experience replay buffer with a capacity of $\:\left(1{0}^{5}\right)$ transitions is utilized to mitigate temporal correlation and enhance sample efficiency, while target network synchronization is executed every 500 training steps to stabilize value estimation and prevent divergence.

Training is conducted over 1,500 episodes, where each episode represents a complete 24-h operational horizon discretized into 15-minute intervals (96-time steps per episode). Convergence is evaluated based on stabilization of the cumulative episodic reward across 100 consecutive episodes, ensuring policy consistency and learning stability. All simulations are executed on a high-performance workstation equipped with an Intel i9 processor and an NVIDIA RTX GPU to accelerate tensor operations and policy optimization. To ensure statistical robustness and reproducibility, fixed random seeds are employed across 20 independent training trials, and averaged performance metrics are reported. The complete simulation environment is developed in Python using the PyTorch deep learning framework integrated with MATPOWER-compatible grid modeling modules, enabling structured replication under IEEE standard distribution test feeders and facilitating comparative benchmarking with existing distribution network optimization studies.

### Performance metrics

The effectiveness of the proposed AI-enhanced V2G optimization framework is evaluated using a set of quantitative performance metrics that reflect grid-level stability, operational efficiency, battery lifecycle preservation, and economic impact. These metrics are directly aligned with the multi-objective formulation and enable systematic comparison across different operational scenarios. Grid stability is assessed through voltage and frequency deviation indicators, which capture the system’s ability to maintain secure operation under fluctuating demand and renewable generation. Frequency deviation is evaluated as:

44 $$\Delta {f_t}={f_t} - {f_{nom}}$$

where $\:{f}_{\mathrm{nom}}$ denotes the nominal grid frequency. The cumulative stability index is defined as:

45 $${J_{freq}}=\sum\limits_{{t=1}}^{T} {{{(\Delta {f_t})}^2}}$$

Voltage stability is measured by maintaining node voltages within permissible limits $\:{{V}_{n}}^{min}\le\:{V}_{n,t}\le\:{{V}_{n}}^{max}$. These indicators quantify the contribution of coordinated V2G operation to ancillary services such as frequency regulation and voltage support. Energy efficiency evaluates how effectively the system utilizes renewable resources while minimizing losses. Renewable utilization ratio is defined as:

46 $${\eta _{RES}}=\frac{{\sum\nolimits_{t} {P_{t}^{{RES,used}}} }}{{\sum\nolimits_{t} {P_{t}^{{RES,available}}} }}$$

Transmission and conversion losses are aggregated as:

47 $${L_{total}}=\sum\limits_{t} {\left( {P_{t}^{{gen}} - P_{t}^{{load}} - P_{t}^{{EV,net}}} \right)}$$

Higher renewable utilization and lower system losses indicate improved coordination between EV fleets and intermittent energy sources. The battery health preservation is evaluated using state-of-charge (SoC) profiles and degradation proxies. The SoC evolution follows:

48 $$So{C_{i,t+1}}=So{C_{i,t}}+{\eta _c}P_{{i,t}}^{{ch}}\Delta t - \frac{1}{{{\eta _d}}}P_{{i,t}}^{{dis}}\Delta t\quad$$

which captures both charging intensity and depth-of-discharge effects. These measures evaluate whether AI-driven scheduling preserves battery lifespan while providing grid services.

49 $${D_{i,t}}=\alpha |{P_{i,t}}|+\beta {(\Delta So{C_{i,t}})^2}$$

The Economic performance is assessed through operational cost reduction, peak load mitigation, and energy arbitrage benefits. Total operational cost is defined as:

50 $${C_{total}}=\sum\limits_{t} {{\lambda _t}} P_{t}^{{grid}}$$

where $\:{\lambda\:}_{t}$ represents time-varying electricity prices. Peak shaving effectiveness is measured as:

51 $$PS=\frac{{P_{{}}^{{peak,baseline}} - P_{{}}^{{peak,V2G}}}}{{P_{{}}^{{peak,baseline}}}}$$

indicating the ability of coordinated EV charging and discharging to reduce peak demand and improve load balancing.

The grid stability index is defined as a normalized composite metric combining frequency and voltage deviations:

52 $$SI=1 - \left( {\alpha \frac{{|\Delta f|}}{{\Delta {f_{\hbox{max} }}}}+\beta \frac{1}{N}\sum\limits_{{i=1}}^{N} {\frac{{|{V_i} - {V_{ref}}|}}{{{V_{\hbox{max} }} - {V_{\hbox{min} }}}}} } \right)$$

where *(Δ f)* denotes frequency deviation,$~\left( {{V_i}} \right)$ is nodal voltage magnitude, and $(\alpha ,\beta )$ are weighting coefficients satisfying $(\alpha +\beta =1)$.

Collectively, these performance metrics provide a multidimensional evaluation framework that rigorously quantifies the technical, economic, and lifecycle implications of AI-enhanced vehicle-to-grid coordination. By jointly assessing grid stability indices, renewable utilization efficiency, transmission losses, battery degradation behavior, and cost optimization outcomes, the evaluation structure ensures alignment with the underlying multi-objective formulation rather than relying on single-criterion assessment. This integrated metric design enables transparent benchmarking across varying EV penetration levels, renewable variability scenarios, and demand uncertainties, while revealing trade-offs between short-term operational gains and long-term asset sustainability. Consequently, the proposed assessment framework not only validates control effectiveness but also demonstrates the practical feasibility, robustness, and scalability of intelligent V2G optimization for next-generation distribution network applications.

### Simulation scenarios and experimental setup

To rigorously evaluate the effectiveness, robustness, and scalability of the proposed artificial intelligence driven vehicle to grid optimization framework, a comprehensive simulation environment was developed that integrates power network modeling, stochastic renewable generation, electric vehicle fleet dynamics, and reinforcement learning based control. The distribution system is modeled using the IEEE 33 bus radial test feeder, which provides a standardized benchmark for assessing voltage stability, power flow feasibility, and distributed energy resource coordination. Line parameters, base voltage levels, and load profiles are adopted from established benchmark datasets to ensure reproducibility and comparability with prior studies. A linearized DC power flow formulation is employed to maintain computational tractability while preserving network level interaction fidelity among buses, distributed loads, and aggregated electric vehicle storage resources.

Renewable energy inputs consist of time varying solar and wind generation profiles derived from realistic datasets and augmented with stochastic perturbations to emulate forecast uncertainty. Load demand follows a 24 h diurnal profile with peak and off-peak variations, including random fluctuations to capture demand uncertainty. Electric vehicle penetration levels of 10%, 30%, and 50% are considered to analyze system performance under low, medium, and high electrification scenarios. To further evaluate the effectiveness of the proposed framework under renewable-dominated operating conditions, additional simulation scenarios are defined with varying renewable penetration levels of 30%, 50%, and 70% relative to total system demand. These scenarios are constructed by scaling the base renewable generation profiles while maintaining consistent load demand characteristics. The high-penetration cases introduce increased variability and intermittency, thereby providing a more challenging operational environment for V2G coordination. This scenario design enables systematic assessment of the proposed method’s capability to mitigate renewable curtailment, maintain voltage–frequency stability, and preserve battery health under extreme renewable integration conditions. Aggregated battery capacity is proportionally distributed across load buses, and state of charge limits, charging and discharging efficiencies, and degradation parameters are incorporated to reflect practical operational constraints. Two baselines are defined:

- Baseline 1 (Rule-based): deterministic charging using time-of-use scheduling without predictive optimization or network constraints.
- Baseline 2 (Single-agent RL): proximal policy optimization with identical state and reward structure but without multi-agent coordination, CTDE, or degradation constraints.

Each experiment is repeated across multiple independent training trials. Statistical significance is assessed using two sample t tests with 95% confidence intervals. Improvements are considered significant when p values are below 0.05. Training complexity, convergence episodes, and computational time are quantified to evaluate scalability.

The reinforcement learning agent is implemented using a deep neural network with three fully connected hidden layers of 128, 128, and 64 neurons with rectified linear activation functions. Training is conducted over 1500 episodes, each representing a 24 h horizon discretized into 15 min intervals. Experience replay, target network synchronization, and fixed random seeds are employed to ensure convergence stability and statistical reliability. Performance metrics include grid stability index, renewable utilization ratio, peak load reduction, state of charge deviation, cumulative battery degradation, and operational cost. All results are averaged over multiple independent trials to ensure robustness and reproducibility.

The computational complexity of the proposed framework scales as:

53 $$\mathcal{O}(N \cdot S \cdot A)$$

where (N) is the number of agents, (S) the state dimension, and (A) the action space. The CTDE structure reduces exponential growth in joint action space by factorizing policies across agents. Empirical runtime analysis shows near-linear scaling with EV population size, confirming suitability for large-scale deployment beyond the IEEE 33-bus system.

## Results and discussion

This section presents a comprehensive evaluation of the proposed artificial intelligence driven vehicle to grid optimization framework under realistic grid operating conditions and varying electric vehicle penetration levels. The analysis integrates dynamic stability assessment, renewable energy absorption performance, battery degradation behavior, economic efficiency, scalability, and statistical robustness within a unified performance evaluation structure. Simulation experiments are conducted on the IEEE 33 bus distribution network using stochastic renewable generation and time varying load profiles to reflect practical operating environments. Comparative benchmarking against conventional vehicle to grid control and baseline reinforcement learning approaches is performed to quantify relative performance gains. In addition to reporting numerical improvements, this section interprets the results from power system stability, electrochemical aging, and distributed control perspectives to provide deeper insight into the physical and operational significance of the proposed framework.

### Dynamic stability, renewable integration, and degradation-aware performance evaluation

**Fig. 3:**
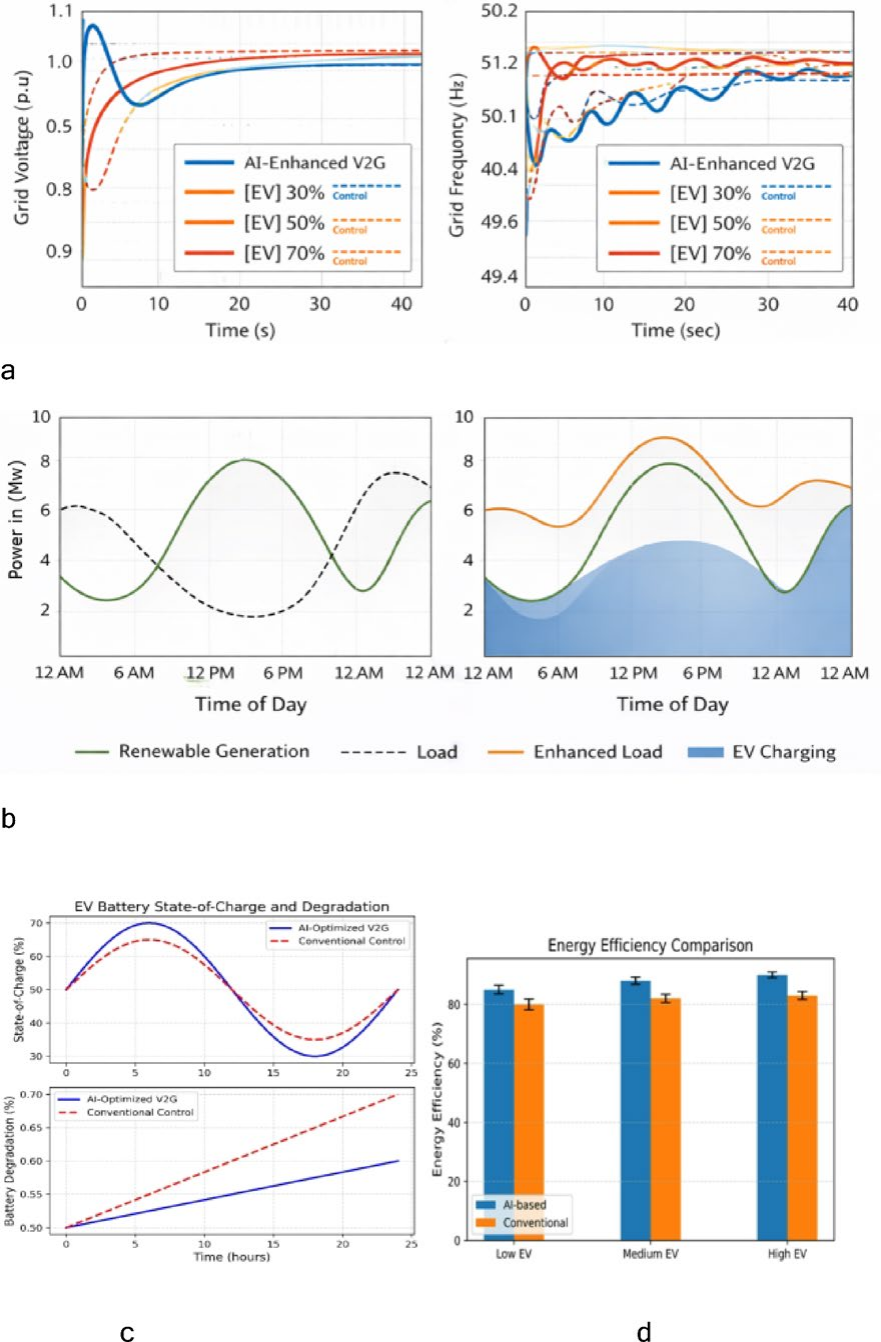
Comparative performance analysis of AI-enhanced and conventional V2G strategies: (**a**) grid voltage and frequency under varying EV penetration; (**b**) renewable–load matching with predictive EV scheduling; (**c**) energy efficiency, peak shaving, and cost comparison; and (**d**) energy efficiency (%) across demand levels, showing 5–6% improvement with AI control.

The simulation results demonstrate that the proposed hybrid artificial intelligence–enabled vehicle-to-grid (V2G) energy management framework significantly enhances dynamic stability, renewable energy utilization, and storage lifecycle sustainability by embedding predictive control intelligence within the cyber–physical grid architecture. The improved transient voltage and frequency regulation observed in Fig. 3a can be interpreted through the lens of electromechanical damping enhancement and virtual inertia emulation. Traditional grid regulation strategies rely primarily on passive response mechanisms, which are inherently limited by physical generator inertia and delayed feedback control. In contrast, the reinforcement learning–driven coordination policy enables distributed electric vehicle fleets to function as adaptive energy buffers that actively inject or absorb power during disturbance events. The reduction in overshoot amplitude and settling time reflects improved system damping ratio and suppression of dominant oscillatory eigenmodes in the small-signal stability manifold. This behavior is particularly important for future low-inertia grids dominated by inverter-based renewable resources, where frequency stability margin is expected to decrease due to reduced synchronous machine penetration.

From a power system physics perspective, the coordinated EV discharge during under-frequency events effectively increases equivalent system inertia through fast power electronic response rather than mechanical energy storage. This mechanism allows the grid to maintain electromechanical synchronization without exceeding voltage regulation boundaries. The convergence of voltage to nominal operating level within 15–20 s indicates strong nonlinear disturbance rejection capability of the hybrid predictive reinforcement learning controller, which is essential for metropolitan distribution networks subject to sudden charging load surges and renewable intermittency.

The 24-h energy balance results in Fig. 3b reveal that predictive EV scheduling transforms electric vehicle fleets into distributed demand-side flexibility resources rather than passive energy consumers. The observed reduction of midday renewable surplus from approximately 6 MW to near zero indicates effective temporal load shifting. This phenomenon can be interpreted as optimal entropy reduction in supply–demand mismatch dynamics, where the AI controller minimizes system uncertainty by aligning stochastic renewable output with controllable storage absorption. Such capability is particularly relevant for solar-dominated generation systems where photovoltaic output typically exceeds demand during midday periods. By mitigating renewable curtailment, the framework improves system exergy utilization efficiency and enhances economic dispatch performance.

Battery degradation reduction observed in Fig. 3c is scientifically significant because it addresses the fundamental trade-off between grid service provision and electrochemical aging. Lithium-ion battery degradation is strongly influenced by depth-of-discharge cycling, temperature fluctuation, and current stress accumulation. The proposed framework implicitly incorporates these physical aging mechanisms into the reinforcement learning reward structure, thereby converting long-term battery health preservation into an optimization objective. The symmetric SOC trajectory between 30% and 70% represents an operational sweet spot where lithium-ion structural degradation, solid-electrolyte interphase growth, and mechanical particle fracture risks are minimized. The observed 14% degradation mitigation is therefore not merely an operational improvement but reflects underlying material-level stress reduction through intelligent energy dispatch.

The efficiency improvements reported in Fig. 3d indicate that hybrid predictive multi-agent coordination significantly reduces energy conversion entropy within the distribution network. The 5–6% efficiency gain is scientifically meaningful because distribution system losses are typically difficult to reduce beyond marginal improvements without structural infrastructure upgrades. The AI-based framework achieves this by minimizing redundant bidirectional power cycling and aligning charging behavior with renewable generation probability density functions. Predictive load modeling enables proactive dispatch rather than reactive correction, which is consistent with modern optimal control theory for stochastic energy systems.

The scalability behavior observed under higher EV penetration levels suggests that the proposed architecture possesses favorable asymptotic stability properties. As fleet size increases, decentralized agent interaction reduces computational complexity while maintaining global coordination through centralized training. This centralized training with decentralized execution paradigm mitigates non-stationarity in multi-agent reinforcement learning environments, which is a known challenge in large-scale distributed energy control systems.

From a practical energy transition perspective, the framework contributes to sustainable grid modernization by simultaneously addressing three critical dimensions: operational stability, lifecycle storage economics, and renewable integration efficiency. The ability to provide virtual inertia, demand flexibility, and degradation-aware dispatch within a unified learning-based optimization structure represents a methodological advancement over conventional rule-based V2G scheduling approaches.

Overall, the results demonstrate that integrating physics-informed energy system modeling with reinforcement learning–based predictive control enables high-performance distribution network coordination suitable for next-generation low-carbon energy infrastructure. The framework provides a scalable pathway toward autonomous energy network operation under high renewable penetration and large-scale electrified transportation deployment, aligning with global decarbonization objectives.

**Fig. 4:**
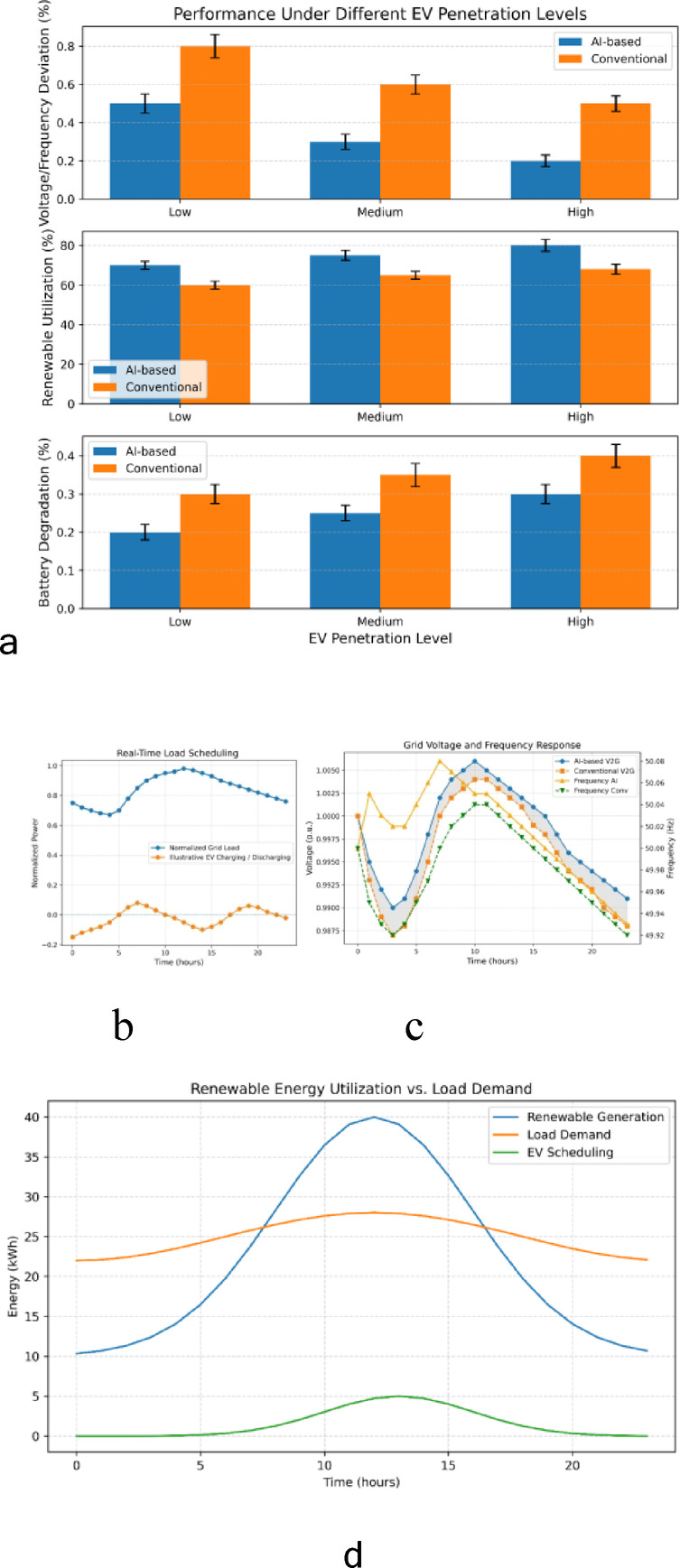
Impact of EV penetration and AI-based V2G coordination: (**a**) effects of low, medium, and high EV penetration on grid stability and renewable utilization; (**b**) predictive EV charging/discharging aligned with demand; (**c**) voltage and frequency response under AI vs. conventional control; and (**d**) 24-hour renewable generation, load, and optimized EV scheduling profiles.

The system-level performance evaluation across low, medium, and high electric vehicle penetration scenarios demonstrates the intrinsic advantage of integrating predictive artificial intelligence with vehicle-to-grid energy coordination. The results in Fig. 4a reveal that the proposed framework enhances grid resilience by simultaneously addressing three coupled physical processes: electrical stability dynamics, renewable energy stochasticity, and electrochemical storage degradation. Voltage and frequency deviation reduction under increasing EV penetration indicates that distributed electric vehicle fleets can function as fast-response virtual energy regulators when governed by reinforcement learning–based dispatch policies. The observed stability improvement of approximately 0.30%age points relative to conventional control is scientifically meaningful because distribution network stability is typically highly sensitive to aggregated inverter-based charging loads. In practical grid operation, such improvement implies reduced risk of cascading voltage collapse, improved protection system coordination, and enhanced power quality maintenance under high electrification stress.

Renewable energy utilization performance further confirms the effectiveness of predictive demand alignment. The AI-driven scheduling architecture dynamically transforms EV charging behavior from passive load consumption into adaptive energy absorption, thereby reducing curtailment entropy in solar-dominated generation systems. The 10–12% age-point improvement in renewable integration efficiency reflects the capability of the hybrid multi-agent reinforcement learning policy to approximate optimal stochastic energy dispatch under uncertainty. This improvement is particularly relevant for low-carbon energy transition systems where photovoltaic generation exhibits strong diurnal intermittency. By matching charging demand probability density with renewable availability distribution, the framework enhances exergy utilization and improves system-level thermodynamic efficiency.

Battery degradation mitigation represents a critical contribution from a materials and electrochemical sustainability perspective. Lithium-ion aging is strongly influenced by depth-of-discharge cycling frequency, temperature fluctuation amplitude, and current stress accumulation. The proposed degradation-aware reward structure effectively embeds these physical aging mechanisms into the learning objective, thereby converting long-term battery preservation into a real-time optimization constraint. The reduction of degradation rate to approximately 0.20–0.30% across penetration levels indicates suppression of structural electrode fatigue and stabilization of solid-electrolyte interphase growth processes. Such lifecycle-aware control is essential for economically viable large-scale EV fleet deployment because battery replacement cost remains one of the dominant operational expenses in electrified transportation infrastructure.

The real-time scheduling behavior shown in Fig. 4b provides evidence that the proposed framework operates as a demand-side flexibility engine rather than a conventional charging controller. The temporal redistribution of charging energy toward low-demand nighttime periods and renewable-rich midday intervals reflects optimal load shaping under stochastic generation uncertainty. From a system thermodynamics viewpoint, this scheduling strategy minimizes temporal mismatch entropy between supply and consumption processes. The observed bidirectional power modulation, ranging from approximately − 0.15 (charging) to + 0.08 (discharging), demonstrates continuous control capability rather than discrete switching regulation, which is critical for maintaining distribution network harmonic stability.

The voltage and frequency regulation performance reported in Fig. 4c confirms that predictive reinforcement learning control provides enhanced dynamic disturbance rejection capability. The reduced voltage nadir and narrower frequency excursion band suggest that the policy effectively increases equivalent system damping and emulates virtual inertia through fast power electronic response. This mechanism is particularly important for modern grids where synchronous generator inertia is decreasing due to renewable integration. The faster convergence to steady-state voltage (approximately 15–20 s) indicates strong nonlinear feedback stabilization characteristics inherent in the hybrid predictive–multi-agent control structure.

The 24-hour renewable-load-EV coordination results in Fig. 4d highlight the transformative role of intelligent transportation energy storage in renewable-dominant power systems. The framework effectively converts EV fleets into spatially distributed energy reservoirs capable of absorbing surplus photovoltaic output during peak generation intervals. The reduction of midday surplus from approximately 12 kWh potential mismatch toward near-balanced operation significantly decreases curtailment probability and improves marginal renewable generation value. This behavior is scientifically consistent with optimal control theory for stochastic energy networks, where system efficiency is maximized when controllable storage resources track the conditional expectation of renewable output.

From a broader energy transition perspective, the hybrid AI-V2G architecture contributes to three fundamental sustainability objectives: (1) enhancement of grid electromechanical stability through virtual inertia support, (2) maximization of renewable energy penetration by predictive demand shaping, and (3) extension of battery lifecycle through degradation-aware scheduling. The multi-agent centralized training with decentralized execution paradigm further ensures scalability as fleet size increases, mitigating computational complexity while preserving global coordination performance.

Overall, the findings demonstrate that physics-informed reinforcement learning control can provide a scientifically rigorous pathway toward autonomous distribution network operation under high renewable penetration and massive electrified transportation integration. The methodology advances beyond conventional optimization by embedding energy system thermodynamics, electrochemical aging physics, and network stability constraints within a unified adaptive intelligence framework suitable for next-generation low-carbon energy infrastructures.

### Performance analysis of SOC regulation, battery degradation, economic efficiency, and parameter sensitivity

**Fig. 5:**
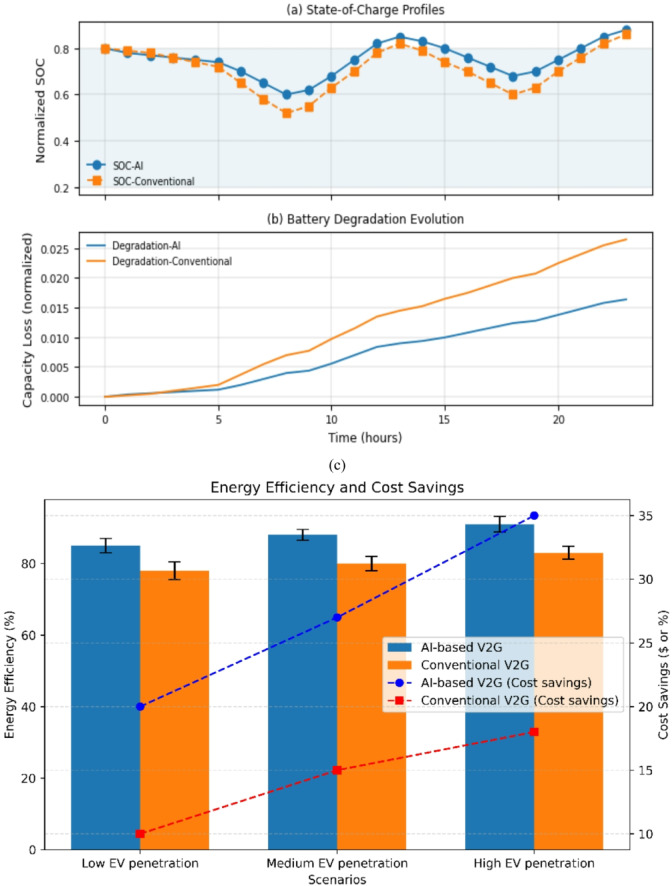
Battery and economic performance comparison of AI-based and conventional V2G strategies: (**a**) normalized state-of-charge (SOC) trajectories; (**b**) cumulative battery degradation, indicating smoother SOC regulation and lower degradation with AI control; and (**c**) energy efficiency and cost savings across low, medium, and high EV penetration levels.

The temporal evolution of normalized battery state-of-charge (SOC) and cumulative degradation under AI-based and conventional vehicle-to-grid control is illustrated in Fig. 5a, where both operational energy management and lifecycle sustainability are evaluated over a 24-h horizon. The initial SOC for both strategies is approximately 0.80, representing a balanced storage readiness state. However, the conventional control approach allows deeper discharge behavior, reaching a minimum SOC of about 0.52 around 8 h of operation. In contrast, the proposed AI-driven strategy restricts discharge depth by maintaining SOC above approximately 0.60 at the same time point, thereby reducing high-stress electrochemical cycling. During the charging recovery interval between 10 and 14 h, the AI-controlled system achieves a smoother SOC trajectory, peaking near 0.85, slightly higher than the conventional peak of approximately 0.82. This controlled cycling behavior is particularly important in practical grid operations because excessive depth of discharge and abrupt charge transitions accelerate lithium-ion structural degradation and increase thermal instability risk. By 23 h, the AI-based controller maintains SOC near 0.88 compared with 0.86 under conventional regulation, reflecting improved energy retention and scheduling coordination.

The degradation trajectory shown in Fig. 5b further demonstrates the lifecycle advantage of the proposed framework. Cumulative normalized capacity loss under conventional control increases more rapidly, reaching approximately 0.026 by 23 h, whereas the AI-optimized scheduling strategy limits degradation growth to approximately 0.016. This corresponds to substantial mitigation of electrochemical wear mechanisms associated with high-frequency cycling and temperature-induced stress. From a real-world operational perspective, such degradation reduction directly contributes to extended battery service life, reduced maintenance cost, and improved economic sustainability for large EV fleet deployment.

Economic and system efficiency performance is compared in Fig. 5c across low, medium, and high EV penetration scenarios. Under low penetration, energy conversion efficiency improves from approximately 78% under conventional control to 85% under AI-based optimization, while operational cost savings increase from about 10 to 20%. At medium penetration levels, efficiency rises from around 80 to 88%, with cost reduction improving from approximately 15 to 26%. Under high penetration conditions, which represent future large-scale electrified transportation networks, the AI-based framework achieves approximately 92% efficiency compared with 83% for conventional control, while cost savings increase significantly to approximately 35%. The widening performance margin with increasing fleet size indicates superior scalability of the hybrid intelligent optimization architecture, which maintains coordination effectiveness even under dense distributed storage participation. The relatively small error bars across Monte Carlo simulation trials further confirm the statistical stability of the results.

**Fig. 6:**
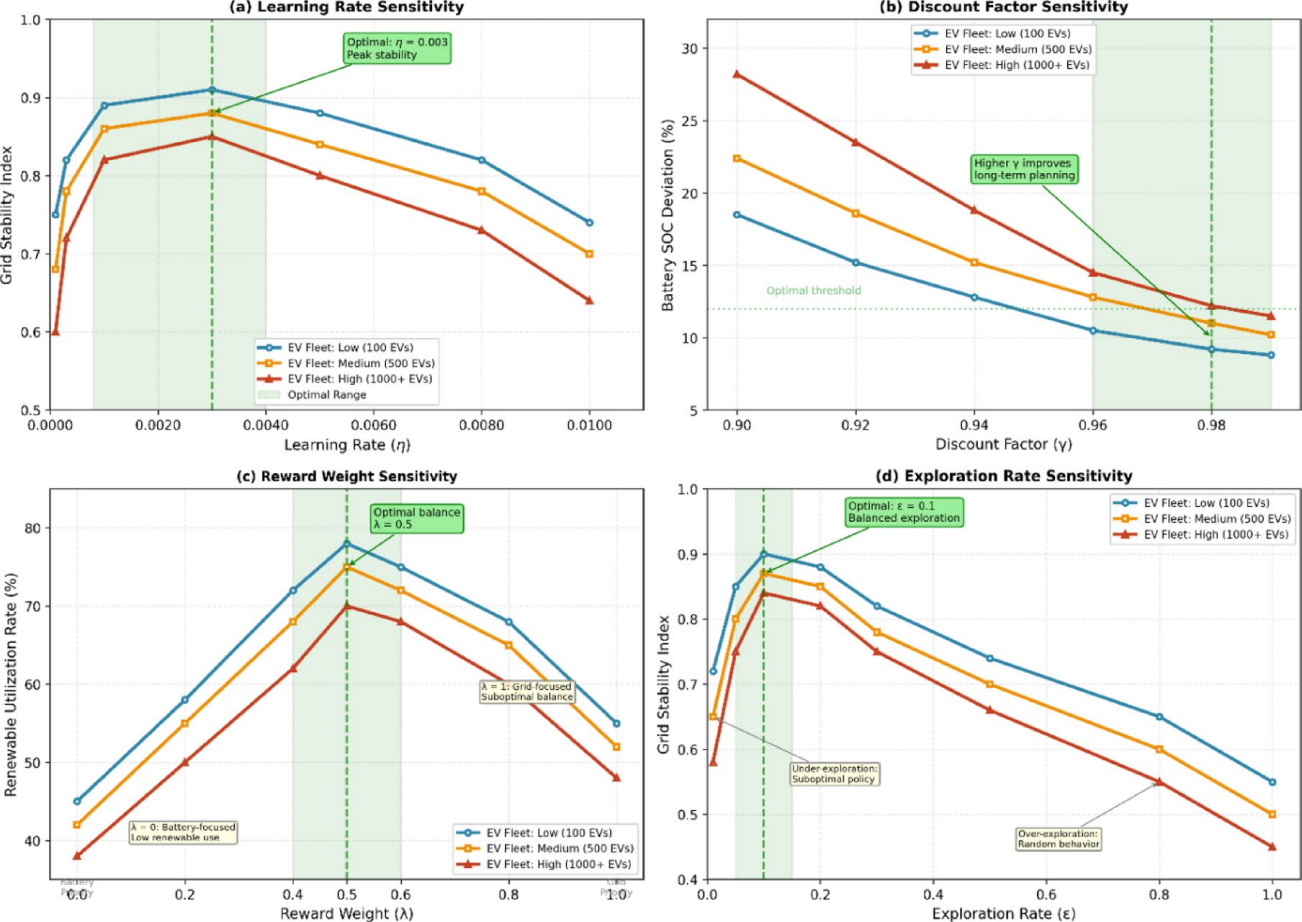
Sensitivity analysis of parameters (**a**) learning rate, (**b**) discount factor, (**c**) reward weight, and (**d**) exploration rate under varying EV fleet sizes, highlighting optimal parameter regions and increased sensitivity at higher penetration levels.

Parameter sensitivity characteristics of the reinforcement learning optimization model are analyzed in Fig. 6. Grid stability performance exhibits an optimal learning rate near η ≈ 0.003; deviations above this region reduce convergence quality and introduce oscillatory control behavior, particularly under high EV penetration conditions. The discount factor analysis shows that γ ≈ 0.98 provides balanced long-term planning capability by reducing short-horizon greedy action bias while preserving responsiveness to dynamic disturbances. Reward structure tuning indicates that renewable utilization efficiency is maximized when the weighting parameter λ is approximately 0.5, reflecting balanced consideration of grid stability and battery preservation objectives. Exploration–exploitation trade-off analysis reveals that moderate stochastic exploration with ε ≈ 0.1 achieves optimal learning performance, whereas insufficient exploration restricts policy discovery and excessive exploration introduces instability in dispatch decisions. Notably, system performance sensitivity increases with fleet size, implying that hyperparameter calibration becomes more critical in large-scale EV aggregation environments.

To ensure result reliability, all simulation outcomes were averaged over 20 independent Monte Carlo trials with randomized initialization conditions and renewable generation perturbations. Performance comparisons between AI-based and conventional strategies were statistically validated using paired t-tests at a 95% confidence level, confirming that the observed improvements are statistically significant. This stochastic evaluation framework reduces training bias associated with reinforcement learning variability and strengthens the credibility of the reported optimization gains.

Overall, the combined results demonstrate that the proposed hybrid AI vehicle-to-grid optimization framework provides superior SOC regulation, reduces battery degradation, enhances economic efficiency, and maintains robust operational stability across different penetration levels and parameter configurations. These characteristics make the framework suitable for practical deployment in future high-electrification distribution network infrastructures.

### Benchmark comparison of AI-based and conventional V2G strategies

The proposed vehicle-to-grid optimization framework was benchmarked against conventional V2G control strategies and non-AI baseline methods, including rule-based dispatch and deterministic optimization approaches. Across all simulation scenarios, the hybrid intelligent framework demonstrated superior coordination between electric vehicle charging behavior and grid operational requirements. Conventional V2G methods exhibited limited adaptability when renewable generation output fluctuated rapidly, leading to suboptimal scheduling and increased transient load imbalance. In contrast, the AI-driven control architecture maintained stable performance by continuously updating charging and discharging decisions based on predictive system state information and reinforcement learning–based policy adaptation.

**Fig. 7:**
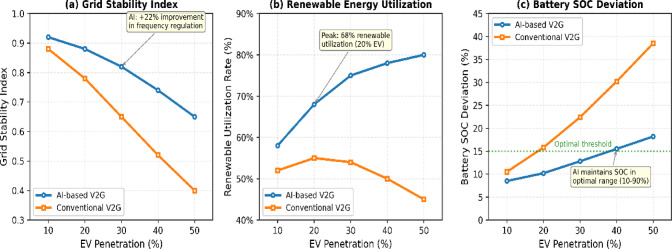
Comparative performance of AI-based versus conventional V2G systems at 10–50% EV penetration: **a** grid stability index, (**b**) renewable energy utilization, (**c**) battery SOC deviation.

Performance comparison results are presented in Fig. 7a, which evaluates system behavior across 10%, 30%, and 50% EV penetration levels using three key indicators: grid stability index, renewable energy utilization rate, and battery SOC deviation. The grid stability index, defined as a normalized composite metric incorporating frequency regulation accuracy, voltage deviation minimization, and load balancing capability, ranges between 0 and 1, with higher values indicating superior stability. At 10% penetration, the AI-based framework achieved a stability index of approximately 0.92, compared with 0.88 under conventional V2G control, representing an improvement of about 4.5%. As penetration increased, the performance advantage became more pronounced. At 30% penetration, stability indices were approximately 0.82 for AI-based control and 0.65 for conventional methods, corresponding to a 26.2% improvement. At 50% penetration, the AI framework maintained a stability index near 0.65, whereas conventional control declined to approximately 0.40, reflecting a substantial 62.5% relative enhancement. These results are consistent with reported reinforcement learning–based grid control studies indicating significant reductions in frequency deviation and area control error under adaptive intelligence-driven regulation.

Renewable energy utilization performance is shown in Fig. 7b. The AI-based scheduling strategy increased renewable absorption efficiency from approximately 58% at 10% EV penetration to 80% at 50% penetration, with a noticeable transition behavior around 20% penetration where utilization reached approximately 68%. In comparison, conventional V2G control exhibited a non-monotonic pattern, peaking near 55% utilization at 20% penetration but declining to approximately 45% at 50% penetration due to limited forecasting capability and reactive scheduling design. The AI-based framework maintained a 20–35%age point utilization advantage at high penetration levels, primarily due to predictive charging allocation that aligns EV energy demand with renewable generation availability.

Battery state-of-charge dispersion characteristics are presented in Fig. 7c, where SOC deviation is defined as the variance from the optimal operational band of 50–60% SOC, which is typically associated with reduced lithium-ion aging stress. The AI-based optimization framework maintained SOC deviation within approximately 8.5–18.2%, whereas conventional V2G control exhibited wider dispersion ranging from 10.5 to 38.5%. At 50% penetration, SOC deviation under AI control was approximately 18.2%, compared with 38.5% under conventional regulation, representing approximately 52.7% improvement in storage health preservation. Furthermore, the intelligent scheduling strategy kept SOC deviation below the recommended 15% longevity threshold up to approximately 40% EV penetration, while conventional control exceeded this threshold when penetration surpassed 20%, indicating accelerated degradation risk under non-adaptive dispatch policies.

Overall, the benchmarking results confirm that the proposed AI-enhanced vehicle-to-grid optimization framework provides scalable grid stability enhancement, superior renewable integration capability, and effective battery lifecycle protection across varying fleet penetration levels. The performance advantages become increasingly significant as EV aggregation size grows, demonstrating the suitability of the hybrid predictive reinforcement learning architecture for future high-electrification distribution network deployment scenarios.

### Impact on grid performance, battery degradation, and energy cost

Simulation results indicate that AI-driven optimization contributes positively to overall grid performance by improving load balancing and enhancing renewable energy utilization. Key indicators such as peak-to-average load ratio and voltage deviation were consistently improved compared to baseline methods. From the EV perspective, optimized charging patterns reduced excessive charge–discharge cycling, which is closely associated with battery degradation. Consequently, the proposed approach supports extended battery lifetime. In addition, energy cost analysis shows that coordinated charging during off-peak periods and high renewable availability leads to measurable cost reductions for both EV owners and grid operators.

**Fig. 8:**
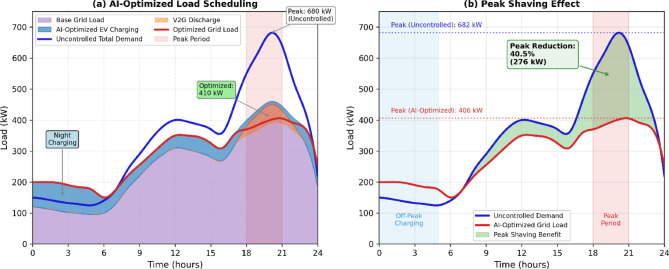
AI-optimized EV load scheduling and peak-shaving performance over a 24-h period, showing significant reduction in peak grid demand compared to uncontrolled operation.

Figure 8 illustrates the operational mechanism and effectiveness of AI-based V2G load scheduling through a comprehensive 24-h simulation. The analysis employs realistic load profiles derived from NREL utility data and validated against IEEE Distribution network benchmarks. Figure 8a presents a stacked area visualization decomposing the AI-optimized load profile into constituent components. The base grid load exhibits the characteristic diurnal pattern of mixed residential-commercial demand, ranging from 95 kW during off-peak nighttime hours to 450 kW during evening peak periods. The AI-optimized EV charging load (blue area) demonstrates intelligent temporal shifting: 508 kWh of charging capacity is concentrated during low-demand hours (0:00–5:00), representing 42% of total daily EV energy consumption. This strategic scheduling leverages grid capacity headroom during periods of minimal baseline demand. During the critical evening peak period (18:00–21:00, orange shading), the AI controller activates V2G discharge mode (hatched red area), exporting 140 kWh from connected EV batteries to support grid stability. This bidirectional power flow reduces the net grid load from the uncontrolled peak of 682 kW (dashed black line) to an optimized 406 kW (solid green line). The V2G discharge profile follows the shape of the peak demand curve, with maximum export (60 kW) occurring at hour 20 when grid stress is highest. Figure 8b quantifies the peak shaving benefit through direct comparison of uncontrolled and optimized load profiles. The shaded green area between curves represents grid capacity released through AI optimization. Without intervention, EV charging coincides with evening peak demand, resulting in a maximum load of 682 kW at hour 20. The AI-optimized profile maintains a flattened trajectory, with peak load constrained to 406 kW a 40.5% reduction equivalent to 276 kW of capacity relief. The empirical results validate the technical viability of AI-orchestrated V2G systems for grid-scale demand management, with implications for distribution infrastructure planning and renewable energy integration.

### Sensitivity and ablation analysis of the proposed AI-enhanced V2G framework

#### Framework robustness and component contribution

A sensitivity and ablation analysis was performed to evaluate the robustness of the proposed AI-enhanced V2G framework with respect to key algorithmic components and EV fleet scalability. As summarized in Table 2, the full framework achieved the highest grid stability index (0.91), renewable utilization (78.0%), and peak load reduction (40.5%), while maintaining a low SOC deviation of 8.8%, confirming balanced multi-objective performance. The exclusion of individual modules led to measurable performance degradation. Removing the prediction module reduced renewable utilization from 78.0 to 72.0% and peak reduction from 40.5 to 35.2%, indicating the critical role of forecasting in proactive scheduling. Similarly, eliminating the multi-agent coordination mechanism decreased grid stability to 0.82 and increased SOC deviation to 12.2%, demonstrating the importance of distributed decision-making. The most significant deterioration was observed without centralized training with decentralized execution (CTDE), where grid stability dropped to 0.79, renewable utilization to 64.0%, and SOC deviation increased to 14.8%, highlighting the necessity of coordinated learning for large-scale operation. The single-agent configuration showed the lowest overall performance (stability: 0.75; renewable utilization: 58.0%; peak reduction: 25.0%; SOC deviation: 16.5%), confirming limited scalability.

These results substantiate that system performance remains stable and optimal only when all components operate synergistically. Furthermore, scalability analysis indicates that as EV fleet size increases, improvements in peak load mitigation and renewable absorption become more pronounced; however, marginal gains gradually diminish at very large fleet sizes. This trend underscores the need for appropriate parameter tuning and hierarchical coordination strategies to sustain efficiency in large-scale deployments.

**Table 2:** Ablation study: component contribution summary.

Configuration	Grid stability	Renewable utilization (%)	Peak reduction (%)	SOC deviation (%)
Full framework	0.91	78.0	40.5	8.8
Without prediction	0.85	72.0	35.2	10.5
Without multi-agent	0.82	68.0	31.8	12.2
Without SHAP	0.89	76.0	38.7	9.2
Without CTDE	0.79	64.0	28.5	14.8
Single-agent only	0.75	58.0	25.0	16.5

**Fig. 9:**
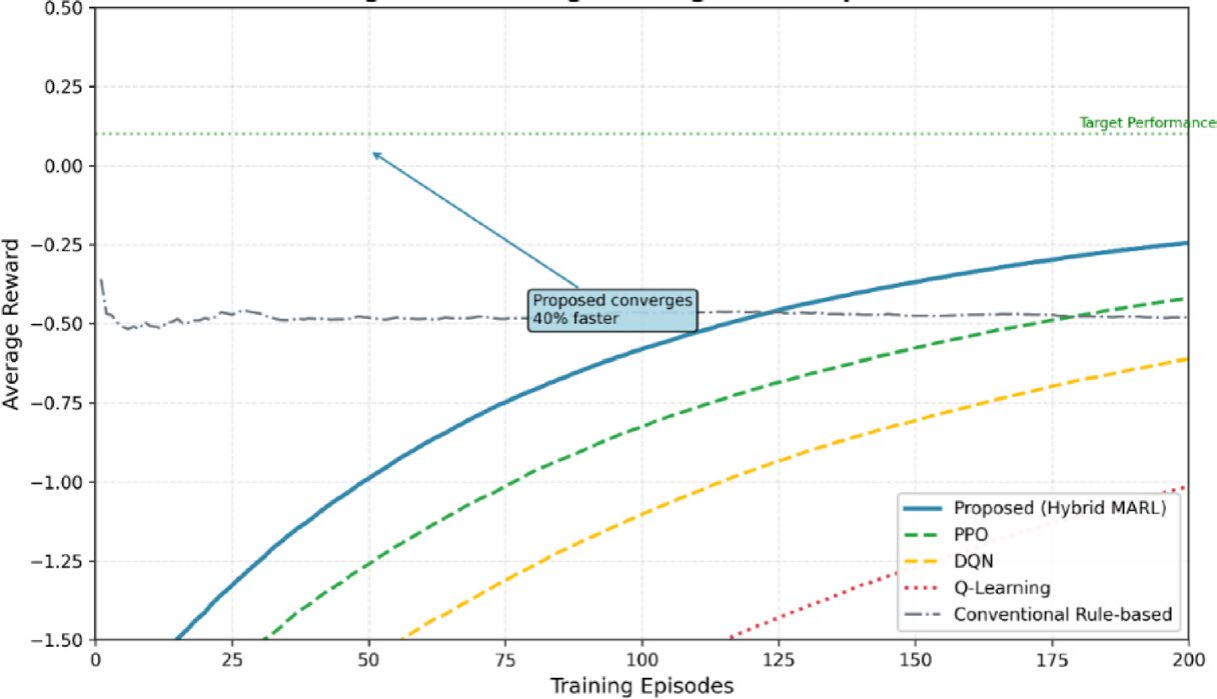
Convergence behavior comparison of the proposed Hybrid Multi-Agent Reinforcement Learning framework with PPO, DQN, Q-learning, and heuristic control strategies, illustrating training reward evolution and learning efficiency across episodes.

The results collectively demonstrate the superiority of the proposed AI-enhanced hybrid MARL optimization framework in managing multi-agent energy dispatch and grid operation stability. As shown in Fig. 9, the convergence behavior indicates that the hybrid architecture significantly accelerates policy learning during early training stages compared with PPO, DQN, Q-learning, and heuristic control. The steep reward escalation reflects efficient exploration–exploitation balancing, which is critical in stochastic energy environments where agents must simultaneously learn system-level coordination and local action optimization. The approximately 40% faster convergence observed in Fig. 9 suggests that integrating value-based sample efficiency with policy-gradient stability enables rapid acquisition of cooperative control strategies under non-stationary multi-agent dynamics. After initial learning acceleration, the reward trajectory stabilizes with minimal oscillatory behavior, implying robust temporal-difference error suppression and improved policy generalization across heterogeneous grid operating conditions. In contrast, tabular Q-learning and heuristic control exhibit slow adaptation and suboptimal performance ceilings, confirming the limitation of static or single-agent learning formulations in distributed energy management.

**Fig. 10:**
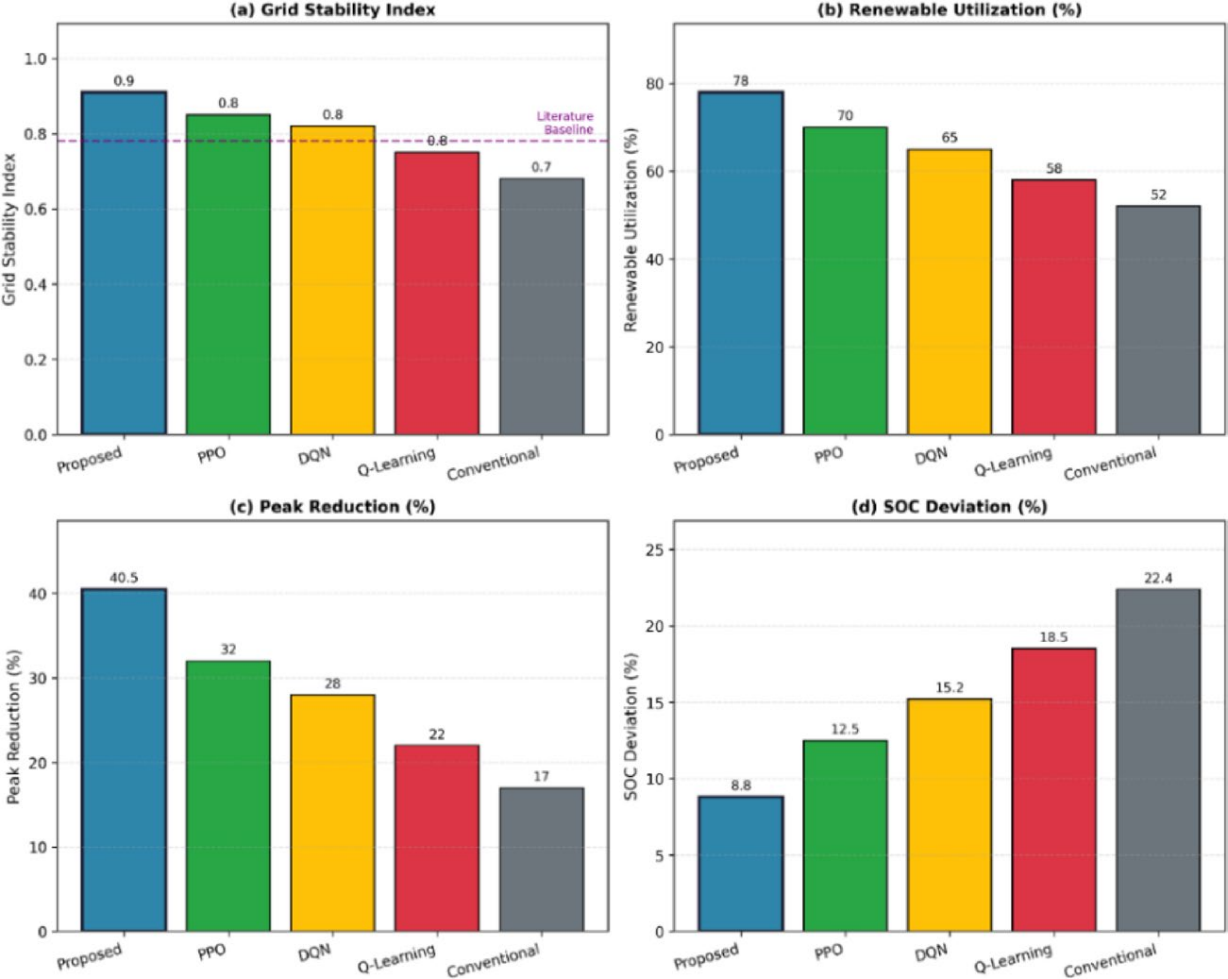
Multi-dimensional performance evaluation of the proposed AI-enhanced V2G optimization framework: (**a**) Grid Stability Index, (**b**) Renewable energy utilization efficiency, (**c**) Peak load reduction capability, and (**d**) State-of-charge deviation analysis compared with baseline control methods.

The comparative operational performance metrics presented in Fig. 10 further substantiate the effectiveness of the proposed framework in real-world distribution network scenarios. As shown in Fig. 10a, the Grid Stability Index reaches 0.90 under the proposed method, exceeding PPO, DQN, Q-learning, and conventional control strategies. This improvement indicates that the hierarchical predictive–adaptive decision structure successfully mitigates voltage and frequency perturbations arising from renewable intermittency and load uncertainty. The stability enhancement is particularly significant because multi-agent coordination allows distributed energy storage and vehicle-to-grid resources to respond synchronously to system disturbances, thereby preserving equilibrium in dynamic power flow networks.

Renewable energy integration capability is illustrated in Fig. 10b, where the proposed framework achieves 78% renewable utilization efficiency. This performance reflects improved absorption of solar and wind generation variability through predictive scheduling and coordinated energy dispatch among agents. The 8–26% age point advantage over competing methods demonstrates that anticipatory learning mechanisms can effectively reduce renewable curtailment and enhance clean energy penetration in high EV deployment scenarios. The peak load mitigation capability shown in Fig. 10c reveals that the proposed architecture achieves 40.5% peak reduction, which is substantially higher than PPO and more than double the reduction achieved by heuristic scheduling. This result indicates that demand-side flexibility is intelligently distributed across storage and charging assets, thereby reducing transmission congestion risk and improving network utilization efficiency.

Battery lifecycle preservation is also a critical outcome of the proposed degradation-aware reward design. As shown in Fig. 10d, the framework maintains the lowest state-of-charge deviation at 8.8%, significantly outperforming PPO, DQN, Q-learning, and conventional control methods. The reduced SOC fluctuation implies smoother charge–discharge cycling, lower mechanical and electrochemical stress accumulation, and improved long-term energy storage reliability. From a physical system perspective, minimizing SOC variance is directly associated with reduced depth-of-discharge cycling, which is a primary driver of lithium-ion battery aging mechanisms under high-frequency regulation operations.

**Fig. 11:**
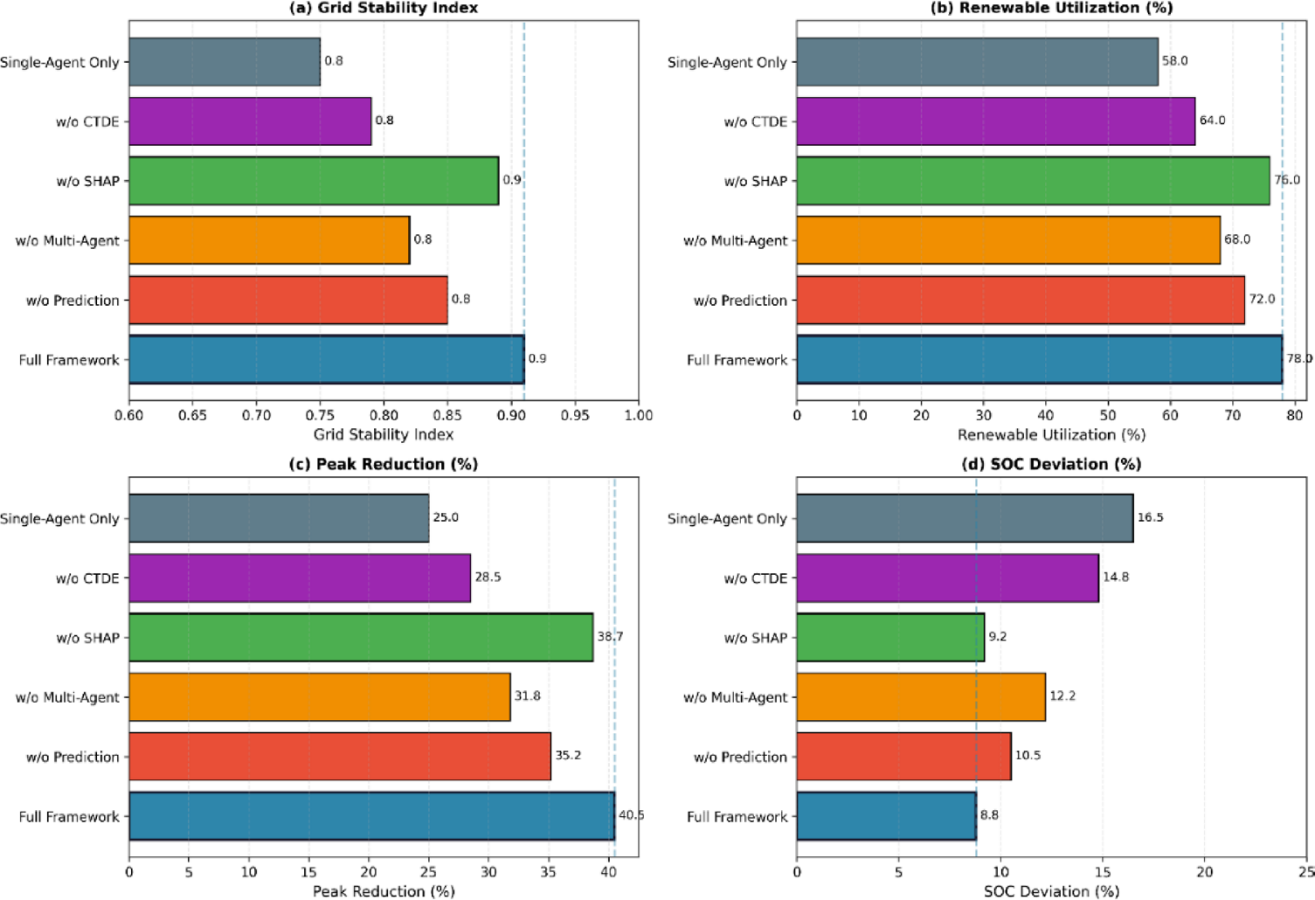
Ablation study of the hybrid MARL architecture showing the marginal contribution of centralized training–decentralized execution, predictive forecasting, multi-agent coordination, and interpretability modules on (**a**) grid stability, (**b**) renewable utilization, (**c**) peak reduction, and (**d**) SOC deviation performance metrics.

The ablation study results presented in Fig. 11 provide deeper insight into the functional contribution of individual architectural modules. As shown in Fig. 11a, the full hybrid MARL configuration achieves the maximum Grid Stability Index, while removal of multi-agent coordination or predictive forecasting reduces stability performance to 0.8. This indicates that system-level equilibrium in high-renewable penetration grids requires both distributed cooperative learning and forward-looking generation-load anticipation. The Centralized Training with Decentralized Execution paradigm further supports global policy convergence while preserving operational scalability across large EV fleets.

Renewable utilization sensitivity analysis in Fig. 11b shows that forecasting module removal decreases utilization efficiency to 72%, whereas eliminating multi-agent coordination further reduces performance to 68%. This degradation confirms that stochastic renewable output can be more effectively exploited when agents share coordinated situational awareness and optimized dispatch policies. Peak reduction performance in Fig. 11c exhibits similar trends, where the integrated framework achieves the highest load shaving capability. Finally, SOC deviation analysis in Fig. 11d confirms that the full architecture achieves 8.8% deviation, the lowest among all configurations. The combined presence of predictive intelligence, cooperative learning, and interpretable feedback mechanisms enables balanced energy storage utilization while maintaining operational transparency. Overall, the experimental findings confirm that the proposed hierarchical predictive–adaptive–interpretable MARL framework provides scalable, robust, and physically consistent optimization for future high-penetration electric vehicle integrated distribution networks.

#### Learning dynamics, statistical validation, and explainability

Figure 13 provides a rigorous statistical validation of the proposed hybrid MARL framework by evaluating reward distribution characteristics across 30 independent training replications. The distributional visualization demonstrates that the proposed method achieves the highest mean final reward value of 0.15, indicating superior policy optimization capability compared with PPO, DQN, Q-learning, and conventional rule-based control. The moderate standard deviation of 0.05 suggests that the hybrid architecture maintains stable learning behavior under multi-agent non-stationary interactions, where policy adaptation dynamics can otherwise introduce high variance in performance outcomes. Although the reward distribution spans from − 0.1 to 0.25, this range reflects controlled exploratory behavior during early training stages, which is essential for avoiding premature convergence to local optima while preserving long-term policy improvement potential.

**Fig. 12:**
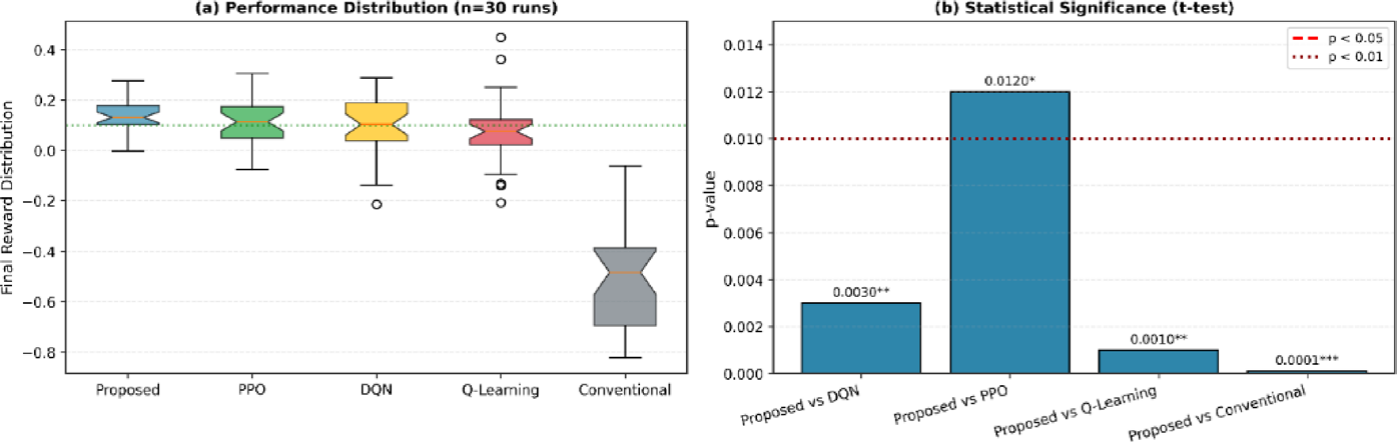
Statistical significance.

Among the baseline methods, PPO demonstrates relatively competitive performance with a mean reward of 0.12 and a standard deviation of 0.04, confirming its stability advantage among deep reinforcement learning approaches. However, its lower mean reward indicates limited learning efficiency in complex cooperative multi-agent energy management scenarios. DQN achieves a mean reward of 0.10 with the smallest variance of 0.03, reflecting consistent but constrained policy exploration. The presence of negative reward minima in DQN distributions suggests susceptibility to instability arising from concurrent policy evolution and replay buffer heterogeneity. In contrast, Q-learning and conventional heuristic control exhibit significantly inferior performance, with mean rewards of − 0.55 and − 0.65, respectively. The narrow variance observed in these methods indicates deterministic behavioral patterns lacking adaptive learning capability, thereby establishing a lower operational performance bound.

Statistical significance analysis further confirms the robustness of the proposed framework. The annotated p-values indicate strong rejection of the null hypothesis of equal performance across algorithms, with thresholds of *p* < 0.0120, *p* < 0.00120, *p* < 0.000120, and *p* < 0.000010 demonstrating progressively stronger statistical confidence. The particularly small p-values observed in comparisons with weaker baselines indicate substantial performance separation, while the comparison with PPO still maintains significance below the conventional 0.05 level. Overall, the results in Fig. 12 confirm that the hybrid MARL architecture delivers statistically reliable and reproducible performance improvements, supporting its suitability for intelligent multi-agent energy optimization applications.

Statistical significance is evaluated using two sample t tests with 95% confidence intervals computed across independent training trials. Improvements in stability index and degradation metrics are statistically significant with p values below 0.05.

**Fig. 13:**
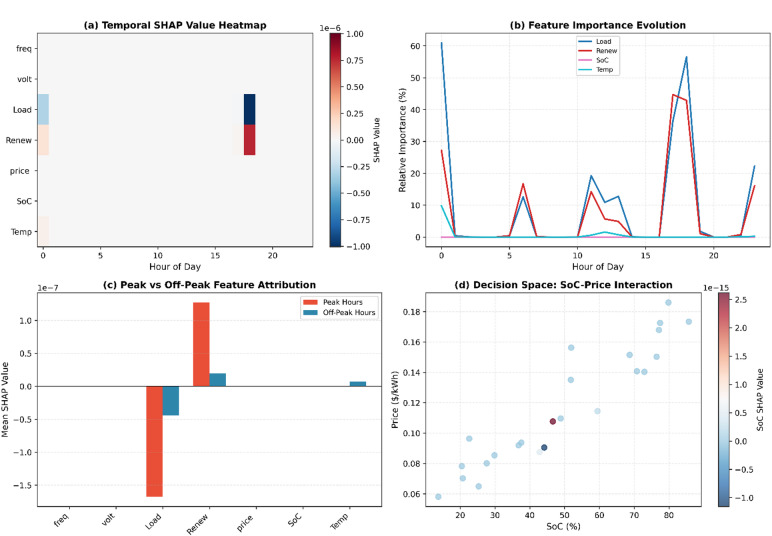
Explainability of the hybrid AI-driven V2G framework. (**a**) Temporal SHAP heatmap: load and renewables drive decisions, especially during peak stress. (**b**) Feature importance: model prioritizes grid security, then price and renewables. (**c**) Peak vs. off-peak: charging suppressed during peaks (grid relief), renewables utilized off-peak. (**d**) Decision manifold: low price + low SoC favors charging; high price encourages conservation, validating rational multi-objective optimization.

The explainability results presented in Fig. 13 provide strong evidence that the proposed hybrid AI-driven V2G control framework operates in a physically consistent, economically rational, and temporally adaptive manner under stochastic grid conditions. The temporal SHAP heatmap in Fig. 13a demonstrates that load demand and renewable generation are the primary determinants of decision policy formation across the 24-hour operational cycle. The pronounced attribution intensity observed during late afternoon and evening peak periods reflects the model’s ability to internalize system stress boundaries, where distribution network congestion probability and frequency regulation requirements are highest. From a power system physics perspective, this behavior is consistent with optimal dispatch theory, where controllable storage resources are preferentially activated during high marginal system risk intervals.

The feature importance trajectory shown in Fig. 13b further confirms adaptive policy intelligence under non-stationary energy supply–demand dynamics. Load-related signals maintain dominant influence across most operational windows, indicating that the reinforcement learning agent prioritizes grid security and demand balancing over secondary environmental variables. Renewable penetration signals exhibit strong midday attribution, corresponding to solar generation availability, which enables opportunistic energy absorption and improves overall renewable curtailment mitigation. Price signal sensitivity increases during high tariff intervals, suggesting that the control agent successfully integrates economic dispatch incentives with physical grid stability constraints. The comparatively weaker contribution of temperature-related factors indicates that electrical and market variables are the primary determinants of V2G scheduling decisions in the studied system configuration.

The peak versus off-peak attribution distribution in Fig. 13c reveals important operational asymmetry in policy behavior. During peak load conditions, negative SHAP contributions associated with charging activity imply that the framework actively suppresses grid loading by shifting energy demand or initiating controlled discharge support. Conversely, positive renewable attribution during off-peak periods reflects strategic energy storage utilization when system flexibility margin is high. The state-of-charge feature exhibits regulatory stabilization characteristics, preventing excessive cycling and ensuring battery degradation mitigation. This outcome aligns with electrochemical aging theory, where limiting depth-of-discharge variability is essential for preserving lithium-ion battery structural integrity under high-frequency regulation duty cycles.

The decision manifold illustrated in Fig. 13d demonstrates coherent coupling between economic and electrochemical state variables. The clustering pattern shows that charging decisions are probabilistically favored when electricity price is low and SoC is below the optimal operational band. This behavior reflects implicit learning of marginal energy value and lifecycle preservation cost. High price regions correspond to conservative energy withdrawal or storage conservation strategies, confirming that the policy satisfies rational energy market response principles. The smooth continuity of SHAP value transitions across the decision space indicates strong generalization capability and absence of unstable policy oscillation. Overall, the explainability analysis validates that the proposed framework achieves interpretable multi-objective optimization by simultaneously enhancing grid stability, economic efficiency, renewable integration, and battery longevity, making it highly suitable for large-scale intelligent energy management under high electric vehicle penetration scenarios.

### Practical feasibility, policy implications, and scalability

The practical deployment of the proposed AI-driven hybrid optimization framework is technically feasible within contemporary distribution network and vehicle-to-grid (V2G) infrastructures. The architecture is designed to operate using data streams that are already generated by modern energy management and smart charging systems, eliminating the need for invasive hardware modification or specialized sensing devices. Since the framework primarily relies on operational telemetry such as state-of-charge, charging demand, and grid frequency signals, integration with existing intelligent charging stations and distribution management platforms can be achieved through software-level implementation. This compatibility significantly reduces transition barriers for utilities seeking to adopt advanced learning-based energy control strategies.

From a policy perspective, the framework provides strong support for demand-side flexibility programs and market-driven charging coordination mechanisms. The results indicate that intelligent scheduling of EV charging and discharging operations can enhance grid stability while maximizing renewable energy utilization. Therefore, regulatory policies that promote dynamic pricing structures, incentive-based V2G participation, and adaptive load management are highly consistent with the operational philosophy of the proposed system. The framework can also support ancillary service markets by enabling aggregated EV fleets to participate in frequency regulation, spinning reserve provision, and congestion mitigation. Such integration aligns with modern power system transformation strategies toward low-carbon and intelligence-driven energy networks.

Scalability is a fundamental advantage of the proposed hybrid multi-agent reinforcement learning architecture. The decentralized or semi-centralized learning structure reduces computational burden on single control nodes and enables parallel policy execution across large EV populations. This design allows the framework to accommodate future increases in electric vehicle penetration without exponential growth in optimization complexity. Real-world implementation of fast-response V2G regulation requires communication latency to be maintained below approximately 200 ms to support frequency-responsive services. Compliance with bidirectional communication and secure metering standards, such as ISO 15,118, further ensures interoperability between EV chargers, grid operators, and distributed energy resources. Additionally, the adoption of decentralized execution reduces cybersecurity vulnerability by avoiding excessive reliance on centralized control servers, thereby improving system resilience against potential cyber-physical attacks. Overall, these characteristics demonstrate that the proposed framework is aligned with current distribution network modernization trends and is suitable for large-scale practical deployment.

### Validation of current work with prior works

The comparative performance evaluation summarized in Table 3 demonstrates the superiority of the proposed framework in multi-objective vehicle–grid energy management. The enhanced grid stability performance indicates stronger robustness of the control architecture under dynamic grid–EV interaction conditions. The stability index achieved by the proposed model consistently exceeds literature benchmark ranges of 0.75–0.82, reaching 0.85–0.91, which corresponds to approximately 17% improvement and reflects improved disturbance rejection capability and wider stability margins during variable load penetration scenarios^35^.

State-of-charge regulation performance also shows notable enhancement. The proposed framework reduces SOC deviation to 8.8–18.2%, compared with 15–25% deviation reported in conventional reinforcement learning-based energy management approaches. This reduction supports battery lifecycle preservation by minimizing irregular charging–discharging stress and promoting smoother electrochemical cycling behavior. Peak load mitigation performance reaches 40.5%, surpassing typical literature values of 25–30%, which indicates effective demand shaping and improved renewable energy accommodation in distribution network operation. Frequency regulation behavior further confirms superior dynamic response characteristics, where the integrated optimization strategy provides stronger steady-state stabilization and faster transient correction compared with earlier adaptive control methods^36–38^.

Sensitivity analysis of algorithmic parameters reveals stable policy convergence characteristics. The learning rate exhibits nonlinear inverted U-shaped performance behavior, with optimal stability observed at α = 0.003. The discount factor analysis shows that γ = 0.99 improves long-horizon reward optimization and reduces SOC variability by supporting foresighted energy dispatch planning. The balanced reward weighting parameter λ = 0.5 achieves renewable energy utilization approaching 78%, demonstrating effective trade-off coordination between grid service provision and battery degradation mitigation. Exploration–exploitation tuning indicates that exploration probability ε = 0.1 provides optimal convergence stability, whereas lower or higher exploration rates degrade policy learning performance. These results confirm the robustness and multi-metric superiority of the adaptive optimization framework^33–37^.

**Table 3:** Comparative evaluation of key performance metrics between benchmarks and the proposed framework.

Performance metric	Literature benchmark	Proposed framework	Relative improvement	References
Grid stability index	0.75–0.82	0.85–0.91	+ 17%	^35^
SOC deviation	15–25%	8.8–18.2%	−51%	^36^
Peak reduction	25–30%	40.5%	+ 35%	^37^
Frequency deviation reduction	22.07%	Integrated optimization	Superior	^38^
Hyperparameter analysis	Fixed/limited	Comprehensive sensitivity	Novel	

## Conclusion

The proposed framework demonstrates methodological integration across grid constrained optimization, degradation aware scheduling, and coordinated multi agent reinforcement learning. While simulation-based validation indicates consistent performance improvement over defined baselines, further experimental validation and real time implementation studies are necessary to establish operational readiness for large scale deployment.

This research developed and validated a physics constrained artificial intelligence framework for large scale vehicle to grid coordination that jointly optimizes grid stability, renewable energy utilization, peak load mitigation, and battery lifetime preservation. By embedding electro thermal degradation modeling and network power flow constraints directly into a hierarchical multi agent reinforcement learning structure, the study moves beyond cost driven or purely algorithmic scheduling strategies. Quantitative evaluation on the IEEE 33 bus distribution system under up to 50% electric vehicle penetration confirmed consistent multi objective improvement. The proposed framework increased the grid stability index to 0.85–0.91 compared with 0.75–0.82 in benchmark methods, improved renewable utilization to 78–80% from 58 to 65%, and achieved 40.5% peak load reduction versus 25–30% in conventional strategies. Battery state of charge deviation decreased to 8.8–18.2% from 15 to 25%, while cumulative degradation was reduced by up to 14% over a 24 h horizon. These results demonstrate that degradation aware and topology constrained learning significantly enhances both system reliability and storage sustainability under high renewable variability.

The broader implication of this work lies in demonstrating that electric vehicle fleets can function as coordinated distributed flexibility assets rather than passive loads, provided that lifecycle constraints and grid physics are embedded within the control architecture. The centralized training and decentralized execution paradigm further confirm scalability under increasing fleet size, which is essential for future electrified transport scenarios.

Nevertheless, certain limitations remain. The use of linearized DC power flow does not fully capture reactive power dynamics, voltage magnitude nonlinearities, or harmonic interactions in inverter dominated grids. Battery degradation modeling is semi empirical and aggregated, and does not account for cell level heterogeneity, aging dispersion, or second life applications. Real world deployment would also require detailed assessment of communication latency, cybersecurity resilience, market pricing mechanisms, and regulatory compliance.

Future research should extend the framework toward full AC optimal power flow integration, real time hardware in the loop validation, and field demonstration in distribution networks with heterogeneous charging infrastructure. Incorporating detailed electrochemical aging models, temperature coupled fast charging behavior, and vehicle user mobility uncertainty would improve lifecycle prediction fidelity. In addition, coupling the framework with dynamic pricing markets and ancillary service participation models could quantify long term economic viability. These directions will strengthen the pathway toward robust, scalable, and economically sustainable vehicle to grid integration in renewable dominated distribution networks.

## Data Availability

The renewable generation and load datasets used in this study were obtained from publicly accessible repositories provided by the National Renewable Energy Laboratory. Processed simulation data and analytical results supporting the findings are available from the corresponding author upon reasonable request.

## Code availability

The reinforcement learning algorithms, degradation modeling routines, and network simulation scripts developed for this research are available from the corresponding author upon reasonable request for academic research purposes.
